# In Ovo Models to Predict Virulence of Highly Pathogenic Avian Influenza H5-Viruses for Chickens and Ducks

**DOI:** 10.3390/v16040563

**Published:** 2024-04-04

**Authors:** Luca Bordes, José L. Gonzales, Sandra Vreman, Sandra Venema, Nadia Portier, Evelien A. Germeraad, Wim H. M. van der Poel, Nancy Beerens

**Affiliations:** 1 Department of Virology, Wageningen Bioveterinary Research, 8221 RA Lelystad, The Netherlands; sandra.vreman@wur.nl (S.V.); sandra.venema@wur.nl (S.V.); n.portier@live.nl (N.P.); nancy.beerens@wur.nl (N.B.); 2 Department of Epidemiology, Bioinformatics & Animal Models, Wageningen Bioveterinary Research, 8221 RA Lelystad, The Netherlands; jose.gonzales@wur.nl

**Keywords:** avian influenza, virulence, ex vivo, in ovo, immunohistochemistry, poultry

## Abstract

Highly pathogenic avian influenza (HPAI) H5-viruses are circulating in wild birds and are repeatedly introduced to poultry causing outbreaks in the Netherlands since 2014. The largest epizootic ever recorded in Europe was caused by HPAI H5N1 clade 2.3.4.4b viruses in the period 2021–2022. The recent H5-clade 2.3.4.4 viruses were found to differ in their virulence for chickens and ducks. Viruses causing only mild disease may remain undetected, increasing the risk of virus spread to other farms, wild birds and mammals. We developed in ovo models to determine the virulence of HPAI viruses for chickens and ducks, which are fast and have low costs. The virulence of five contemporary H5-viruses was compared studying replication rate, average time to death and virus spread in the embryo. Remarkable differences in virulence were observed between H5-viruses and between poultry species. The H5N1-2021 virus was found to have a fast replication rate in both the chicken and duck in ovo models, but a slower systemic virus dissemination compared to three other H5-clade 2.3.4.4b viruses. The results show the potential of in ovo models to quickly determine the virulence of novel HPAI viruses, and study potential virulence factors which can help to better guide the surveillance in poultry.

## 1. Introduction

Eight genetic subgroups (a–h) of HPAI clade 2.3.4.4 H5-viruses have emerged and several of these subgroups have caused repeated outbreaks in Europe resulting in devastating losses to the poultry sector [1,2,3,4,5], mass mortality events among wild birds [6] and occasionally introduction into mammalian species [7,8,9]. HPAI viruses cause severe disease and high mortality in Galliformes poultry, such as chickens and turkeys [10]. However, mortality and morbidity in Anseriformes poultry, such as Pekin ducks and domestic geese is highly dependent on the genetic composition of the virus [11,12]. Thus, the virulence of HPAI H5-viruses may depend on the specific poultry species and genetic characteristics of the virus. Viruses that are less virulent for ducks may not cause overt clinical disease and remain undetected in infected farms, thereby increasing the risk for spread of the virus, spill backs to wild birds and spill overs to mammals. The virulence of novel HPAI viruses is currently assessed by intravenous pathogenicity index (IVPI) which is performed in six-week-old chickens [13]. HPAI viruses have an IVPI score of greater than 1.2 in 6-week-old chickens, according to the World Organization for Animal Health (WOAH) criteria [13]. The major determinant of virulence is the HA gene, although other genes may contribute to increased pathogenicity [14,15]. For subtype H5 viruses, the HPAI phenotype correlates with the presence of multiple basic amino acids at the endoproteolytic cleavage site (CS) of the HA protein [16,17,18]. The IVPI experiments take long to perform, are associated with high costs, and are making use of animals. Furthermore, the chicken IVPI is mainly used for discriminating between low pathogenic avian influenza (LPAI) and HPAI and only a minor selection of the circulating HPAI viruses are currently assessed for virulence in chickens and virulence for ducks is not regularly tested.

Virulence in chickens and Pekin ducks has been previously investigated by IVPI for the clade 2.3.4.4c H5N8-2014, clade 2.3.4.4b H5N8-2016, H5N6-2017 and H5N8-2020 HPAI viruses isolated from index farms in the Netherlands [19,20]. The highest IVPI score of 3.00 was measured for all viruses in chickens, whereas striking differences were observed in Pekin ducks. The virulence of the H5N8-2016 and H5N6-2017 viruses (IVPI around 3.00) was higher than that of the H5N8-2014 and H5N8-2020 virus (IVPI 1.8). This is in line with reports on virulence in Anseriformes poultry which noted a frequent increase in mortality and morbidity on infected farms during the H5N8-2016 and H5N6-2017 epizootics, while mortality and morbidity was limited or absent in some cases during the H5N8-2014 and H5N8-2020 epizootics [21,22,23,24]. The 2020 HPAI epizootic in the Netherlands was followed by the H5N1-2021 HPAI epizootic, which was the largest ever recorded in Europe [25]. In some cases, limited mortality and morbidity was reported in Anseriformes poultry, suggesting reduced virulence in duck species similar to that of the H5N8-2014 and H5N8-2020 epizootics. It is currently unknown how the virulence of the H5N1-2021 HPAI virus in Galliformes and Anseriformes poultry compares to the virulence of previous HPAI H5 clade 2.3.4.4 viruses.

In this study, we aim to develop ex vivo models to determine the virulence of emerging HPAI viruses for poultry species. Ex vivo models can be rapidly implemented during emergence of novel HPAI viruses and could provide more accurate discrimination between HPAI virus virulence than the IVPI models. We developed a chicken-and-duck in ovo model to determine the virulence of five recent HPAI H5-viruses isolated from index farms in the Netherlands (H5N8-2014, H5N8-2016, H5N6-2017, H5N8-2020 and H5N1-2021). Methods were developed to analyze replication of the virus in the allantoic fluid, time to death of the embryos and virus antigen score in several organs of the embryos. Virus replication is a key aspect of virulence and was suggested to influence the severity of HPAI epizootics [26]. Furthermore, virus adaptations to replicate more efficiently in specific host species modify the virulence and are known to occur frequently [27,28]. In this study, we developed a method to sample the allantoic fluid of an individual embryonated egg over time to study virus replication. The survival of animals is an important readout for HPAI virus virulence, and its complement, mortality, is used as an indicator for virus detection in commercial poultry farms. Previous studies have indicated a large difference in the time to death of embryonated chicken eggs infected with low pathogenic avian influenza (LPAI) compared to HPAI viruses [29]. Therefore, the in ovo time to death after HPAI virus infection could be a useful model to study differences in virulence. Currently, candling is used as a method to determine the time to death of embryonated eggs, but we developed a method using a heart rate monitor to determine with higher accuracy the time to death of chicken and duck embryos infected with HPAI viruses. Lastly, the tissue tropism of the virus can provide useful information on virus dissemination and differences in virulence between HPAI viruses. In ovo models can be used to investigate in situ virus replication of HPAI viruses and may provide valuable information on disease progression in poultry [29,30,31]. The results of the in ovo model were compared to previously obtained IVPI results, and the IVPI for the H5N1-2021 virus was performed in this study. The in ovo models were able to identify differences in virulence between the recent HPAI H5-viruses for chickens and ducks. Monitoring changes in HPAI virulence for poultry allows for the annotation of genetic virulence factors, surveillance of host drift and rapid identification of HPAI infected animals with or without overt clinical disease. The results of this study show that ex vivo models are highly valuable tools for risk assessment of HPAI viruses, allowing for a more targeted approach for HPAI surveillance benefiting both public health and animal health.

## 2. Materials and Methods

### 2.1. Virus Isolation, Sequencing and Phylogenetic Analysis

All viruses used in this study were obtained from the index cases in commercial poultry farms during the corresponding epizootics in the Netherlands. Viruses were isolated by two passages in 9- to 11-day-old specific pathogen free (SPF) embryonated chicken eggs (ECEs) (Royal GD, Deventer, the Netherlands). Full genome sequencing of the E2 egg passage was performed as described earlier [32]. In addition, RNA isolated directly from the swab material was sequenced. In short, virus RNA was isolated using the High Pure Viral RNA kit (Roche, Basel, Switzerland), amplified using universal eight segment primers and directly sequenced at high coverage (average > 1000 per nucleotide position) using Illumina DNA Prep and Illumina MiSeq 150PE sequencing. The CLC Genomics Workbench extension ViralProfiler-Workflow (Qiagen, Germany) was used to map the reads to a reference set of genomes. Consensus sequences were generated and uploaded to GISAID. To indicate possible adaptations acquired during passaging, differences detected between the sequences of the swab materials and the second egg passage are listed in Table 1. Only minor differences between the sequences of the swabs and egg passages were detected, and no mutations on positions associated with virulence changes were identified. Median egg infective dose (EID50) of the virus isolates was determined by end-point titration in 9- to 11-day-old SPF ECEs. The virus isolates were titrated in triplicate on different days and EID50 titers were calculated using Reed and Muench [33]. Phylogenetic analysis of the complete genome sequences was performed for each genome segment separately, aligning the virus sequences using MAFFT v7.475 [34], reconstructing the phylogeny using maximum likelihood (ML) analysis with IQ-TREE software v2.0.3 and 1000 bootstrap replicates [35]. ML tree was visualized using the R package ggtree [36].

### 2.2. IVPI in Chickens and Ducks

The IVPI was determined for the H5N1-2021 virus isolate (EPI_ISL_9856775 A/chicken/Netherlands/21037287-006010/2021) in chickens according to the WOAH standard procedure [13]. The IVPI was determined for the same virus isolate in Pekin ducks using a similar procedure, as described previously [20]. Ten six-week-old SPF White Leghorn chickens were acquired from Royal GD (Deventer, the Netherlands) and ten six-week-old Pekin ducks were obtained from a commercial breeding farm. Both male and female animals were used in the study. To exclude ongoing or previous avian influenza virus (AIV) infection in the Pekin ducks, in-house ELISA on sera, quantitative real-time RT–PCR targeting the matrix gene (M-PCR) on swabs from the day of arrival and after a 7-day acclimatization period was performed as previously described [10,32]. Allantoic fluid (stock 9.79 log_10_ EID50/mL) was diluted 1:10 in phosphate-buffered saline (PBS) and 0.1 mL per animal was inoculated intravenously. The animals were monitored for clinical signs and mortality for 10 days and IVPI scores were calculated.

### 2.3. Chicken and Duck In Ovo Model

SPF ECEs were acquired from Royal GD (Deventer, the Netherlands) and embryonated Pekin duck eggs (EDEs) were obtained from a commercial breeding farm. Both ECEs and EDEs were incubated at 37 °C, 65% humidity. ECEs and EDEs were collected from the same breeding pair for the duration of an experiment. To exclude the presence of a previous parental AIV infection, egg yolk was harvested from five EDEs from each breeding pair and subsequently tested for presence of maternal antibodies using the IDEXX AIV Ab ELISA (Hoofddorp, The Netherlands) according to the manufacturer’s instructions. After 9 days of incubation, ECEs and EDEs were candled daily and unfertilized or deceased eggs were removed. After 14 days incubation a dosage of 10^5^ EID50 virus particles diluted in 0.2 mL of 2.95% Tryptose Phosphate Broth (TPB) was used to inoculate the ECEs and EDEs via the allantoic sac for all experiments. Five ECEs and five EDEs were used per virus isolate or control group (inoculated with 0.2 mL TPB).

To determine virus replication in the allantoic fluid a 26-gauge cannula (BD Neoflon™, Singapore) was attached to the ECEs and EDEs allantoic sac (Figure S1). The next day (14-day-old) eggs were candled to remove deceased eggs and proper attachment of the cannula was confirmed by drawing allantoic fluid via the cannula. Subsequently, the five virus isolates were inoculated via the cannula and carefully resuspended in the egg allantoic fluid. TPB was used for the control group. Between 0 and 15 h post infection (p.i.), five samples of 0.2 mL allantoic fluid were collected, AIV RNA was extracted using the MagNa Pure 96 system (Roche, Basel, Switzerland) and detected by M-PCR. Standard curves were used to calculate EID50 equivalents. The experiment was performed in triplicate.

To determine the mean time to death of the 14-day-old ECEs and EDEs after inoculation with the five virus isolates, the eggs were monitored first at 12 h p.i. and from 24 h p.i. onwards hourly using the buddy Mk2 digital egg heart rate monitor according to the manufacturer instructions (Avitronics, Cornwall, UK). AIV infection was confirmed by M-PCR as described above. Deceased eggs at 12 h p.i. were marked as right-censored in the time to death analysis due to technical issues (dropout rate off 5%). The experiment was performed in triplicate.

In a separate experiment to determine the in ovo tissue tropism 14-day-old chicken and duck embryos inoculated with the five virus isolates or 0.2 mL TPB for the control group were humanely killed by cessation of circulation and decapitation at 20 h, 24 h and 28 h post infection. Five embryos were used per virus isolate and time point. The chorioallantoic membranes and embryos were fixated whole in 10% neutral buffered formalin for a minimum of 48 h. Tissues were processed and evaluated for AIV nucleoprotein expression with immunohistochemistry (IHC) as described previously [37]. IHC scoring was performed under supervision of a board certified pathologist and scoring method is described in Table S5.

### 2.4. Statistics

For the in ovo time to death analysis, a Cox proportional model was used where time to death was modeled as a function of virus isolate and host species. To assess the effect of using three repetitions per species and virus, a model was fitted including the “repetition identifier” within a clustering variable. The simple Cox model excluding the cluster effect was the best fit and was used for further analysis. Hazard rates were compared between virus isolates within the same host species. For pairwise comparisons a Tukey correction was applied to adjust *p*-values for multiple comparisons [38].

Replication rates were assessed by fitting a linear mixed-regression broken stick model which models virus replication over time in two phases. The first phase consisted of the first 6 h of virus replication and the second phase between 6 h and 15 h of virus replication. A linear mixed model (LMM) with individual egg as random intercept and virus as random slope was the best fitting model for this data set and was used for further analysis. The model adjusts for the initial virus load at 0 h p.i. in time. Pairwise comparisons were performed applying a Tukey correction for multiple comparisons.

Finally, factor analysis was used to reduce the number of assessed variables by grouping IHC scores from organs based on the statistical correlation. Sample adequacy for factor analysis was determined using the Kaiser–Meyer–Olkin test. The best fitting model of the factor analysis resulted in grouping all IHC scores in a single factor. This factor is described by a calculated score in time, which represents an overall measure of the systemic dissemination of the virus in the embryo. The factor values were then assessed by fitting a regression model to compare systemic dissemination of the five HPAI H5-viruses within each host species. Pairwise comparisons were made applying a Tukey correction for multiple comparisons.

Statistical analysis was performed using the statistical software R, the LMM was fitted using the package lme4, time to death analysis was performed using the library survival, and the factor analysis was performed using the library Hmisc [39,40,41,42]. Post hoc tests for pairwise comparisons were performed using the package emmeans [43].

## 3. Results

### 3.1. Mild Virulence of HPAI H5N1-2021 Virus in Ducks Compared to Chickens

**Figure 1:**
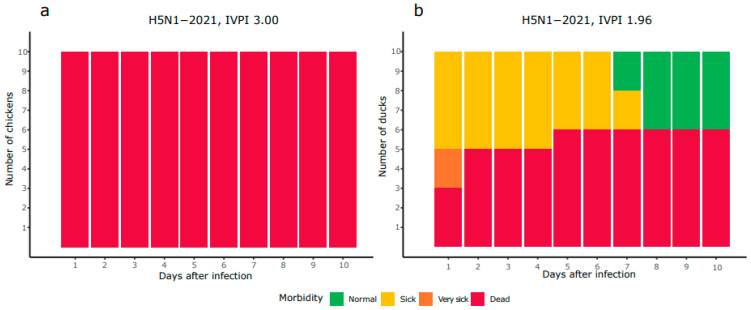
Morbidity and mortality of chickens (**a**) and Pekin ducks (**b**) inoculated intravenously with H5N1-2021.

The virulence of the HPAI H5N1-2021 virus was determined in chickens and Pekin ducks in IVPI experiments. Ten chickens and ducks were intravenously inoculated with the virus, and subsequently monitored for 10 days for clinical signs and mortality. The mortality in chickens was 100% after 24 h, while the mortality in Pekin ducks was 60% after 10 days (Figure 1). All animals showed symptoms related to HPAI infection such as lethargy, tremors, paralysis and respiratory signs. In chickens, the highest IVPI score of 3.00 was reached, while the IVPI score of 1.96 in Pekin ducks was lower. The IVPI score of the HPAI H5N1-2021 virus was compared to that obtained for four viruses from other HPAI epizootic waves in the Netherlands (Table 2, data obtained in the same department). Phylogenetic analysis of these viruses shows that the H5N8-2014 virus belongs to H5 clade 2.3.4.4c, whereas H5N8-2016, H5N6-2017, H5N8-2020 and H5N1-2021 are H5 clade 2.3.4.4b viruses (Figure S2). The four HPAI viruses classified as clade 2.3.4.4b primarily differed on segments PB2, PB1 and NA, while smaller differences were observed on NP, NS and PA and minimal differences were found on HA and MP. The IVPI score in chickens for all viruses was approximately 3, and for the H5N8-2016 and H5N6-2017 viruses, a score of 3 was also measured in Pekin ducks (Table 2). However, the IVPI score of the H5N1-2021 virus in Pekin ducks (1.96) resembles that of the H5N8-2014 and H5N8-2020 viruses.

### 3.2. Replication Rate of HPAI H5-Viruses in Embryonated Chicken and Duck Eggs

**Figure 2:**
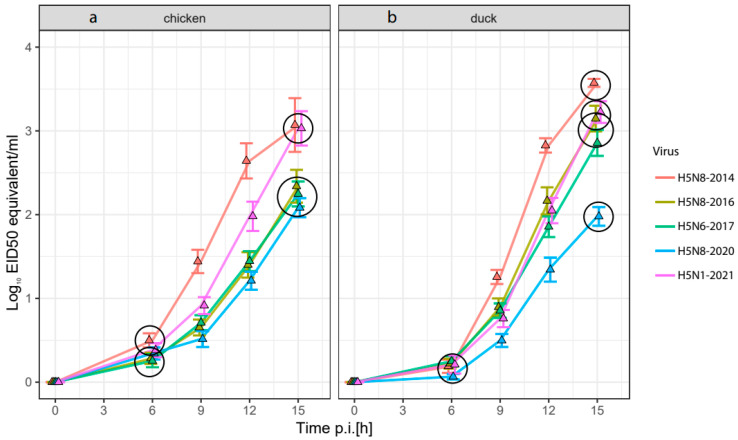
Increase in virus titer from the inoculation dose (0 h time point) of five HPAI viruses in the allantoic fluid of embryonated eggs of (**a**) chickens and (**b**) Pekin ducks over time. Measured by RT-PCR and adjusted to mean log_10_ EID50/mL (depicted by the triangles). Standard error depicted by error bars. Circles indicate significant differences in the replication rate from 0 to 6 h and from 6 to 15 h post infection (*p* < 0.05). In the chicken model, two clusters are observed during both time frames: Cluster 1 (H5N8-2014 and H5N1-2021), followed by Cluster 2 (H5N8-2016, H5N6-2017 and H5N8-2020). In the duck model, multiple overlapping clusters are observed during the 6 to 15 h time frame: Cluster 1 (H5N8-2014), Cluster 2 (H5N1-2021 and H5N8-2016), Cluster 3 (H5N8-2016 and H5N6-2017), and lastly Cluster 4 (H5N8-2020). In the 0 to 6 h time frame, only one cluster including all viruses was observed in the duck in ovo model.

Virus replication of the five selected HPAI H5-viruses was measured over time in the allantoic fluid of embryonated chicken and Pekin duck eggs. A novel technique was used in which allantoic fluid was sampled from individual eggs over time using cannulas. Virus replication was measured using M-PCR (Figure 2). Virus replication rates were analyzed separately for the 0 to 6 h interval and 6 to 15 h interval using a linear regression broken stick model. This type of analysis was selected because the replication rate of the five selected HPAI H5-viruses follows an initial startup phase followed by a phase of rapid increase in the release of the virus (Figure 2, Tables S1 and S2). In the 0 to 6 h time interval, two clusters of viruses could be observed in the chicken in ovo model: the first cluster contained the faster replicating H5-viruses H5N8-2014 and H5N1-2021, and the second cluster contained the slower replicating H5N8-2016, H5N6-2017 and H5N8-2020 viruses. The replication rates of the subsequent clusters were also significantly different during the 6 to 15 h time interval with larger differences between the replication rates.

There were no significant differences in the duck model during the 0 to 6 h time interval. During the 6 to 15 h time interval, the H5N8-2014 virus was also the fastest replicating virus in the duck in ovo model, but the replication rate of the H5N1-2021 virus was lower than the replication rate of the H5N8-2014 virus. Furthermore, the H5N8-2016 and H5N6-2017 viruses replicated significantly faster than the H5N8-2020 virus in the duck allantoic fluid, which was not the case in the chicken in ovo model. The H5N8-2020 virus replicated significantly slower than the other four viruses in duck eggs. The H5N8-2016 and H5N6-2017 viruses shifted from the slower replicating cluster in the chicken model to a faster replicating cluster in the duck model, while the other three HPAI viruses did not move to another cluster or shifted to a lower virulence cluster in the duck in ovo model.

### 3.3. Time to Death of Chicken and Duck Embryos after Inoculation with HPAI H5-Viruses

**Figure 3:**
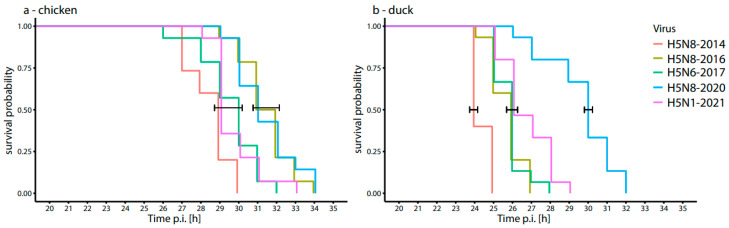
Survival probabilities of embryonated chicken (**a**) and duck (**b**) eggs infected with five different HPAI viruses (*n* = 5 per group). Significant differences are marked by the brackets (*p* < 0.05).

The time to death of both chicken and duck embryos after inoculation with the five HPAI viruses was followed using a heart rate monitor until all ECEs and EDEs were deceased (Figure 3, Tables S3 and S4). In both models, the embryos infected with H5N8-2014 had the shortest time to death, while the embryos infected with H5N8-2020 had the longest time to death, which matches with our findings from the virus replication rates in the allantoic fluid (Figure 2). Two significantly different clusters could be observed in the chicken in ovo model. The subgroup with relatively short time to death includes H5N8-2014, H5N6-2017 and H5N1-2021, while a significantly longer time to death is observed for the H5N8-2016 and H5N8-2020 viruses. In the duck in ovo, model the H5N8-2014 virus has significantly shorter time to death compared to the other viruses, whereas the H5N8-2016, H5N6-2017 and H5N1-2021 viruses have a significantly shorter time to death compared to the H5N8-2020 virus. The largest difference in time to death between chickens and ducks was observed for the H5N8-2016 HPAI virus.

### 3.4. Systemic Virus Spread in Chicken and Duck Embryos after Infection with HPAI-H5 Viruses

**Figure 4:**
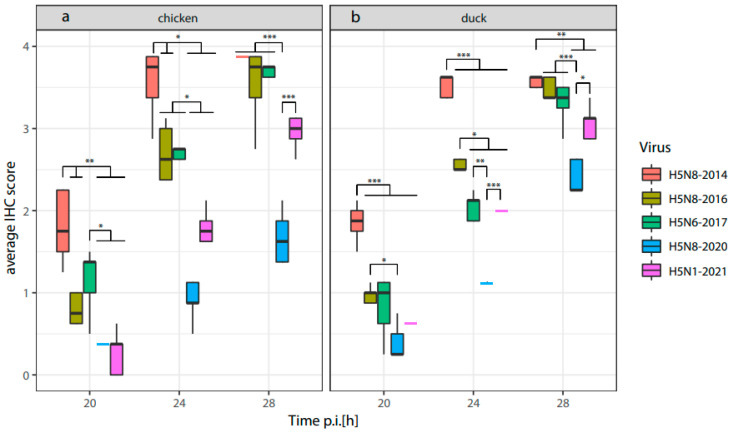
Average virus antigen score (immunohistochemistry) over time in tissues of embryonated chicken (**a**) and duck (**b**) eggs infected with five HPAI H5-viruses. If a boxplot could not be generated the mean is indicated by a colored line. Investigated tissues: Cerebrum, conchae submucosa, conchae epithelium, lung, heart, liver, intestine and chorion allantoic membrane. *N* = 5 per species and virus,* *p* < 0.05, ** *p* < 0.01 and *** *p* < 0.001.

To determine the spread of the virus in the embryos of chickens and ducks, we measured the viral antigen score in eight selected tissues (Cerebrum, conchae submucosa, conchae epithelium, lung, heart, liver, intestine and chorion allantoic membrane) at 20, 24 and 28 h post infection (Figure S3). A factor analysis indicated that virus antigen expression in all tissues followed the same trend. Therefore, the average virus antigen score was calculated for each time point (Figure 4, Tables S6 and S7). At 20 h and 24 h p.i., the virus antigen expression was most abundant in ECEs and EDEs infected with the H5N8-2014 HPAI virus. ECEs and EDEs infected with the H5N8-2020 HPAI virus showed the lowest amount of virus antigen expression at any timepoint compared to the other four HPAI H5-viruses. This matches with our findings from the virus replication rates in the allantoic fluid and the time to death of ECEs and EDEs.

At 24 h p.i., significantly higher virus antigen scores are detected for the H5N8-2016 and H5N6-2017 HPAI viruses compared to the H5N8-2020 and H5N1-2021 HPAI viruses in the ECEs. Significantly higher virus antigen expression was detected in EDEs infected with the H5N8-2016 HPAI virus at 24 h p.i. compared to the virus antigen expression of EDEs infected with H5N6-2017, H5N8-2020 and H5N1-2021. However, in both ECEs and EDEs, the H5N1-2021 virus antigen expression did not increase to the levels that could be expected from the fast replication in the allantoic fluid and short time to death determined in the previous in ovo measurements. This may indicate a delayed systemic dissemination of the H5N1-2021 HPAI virus for chickens and, to a lesser extent, ducks.

### 3.5. Tissue-Specific Tropism of HPAI H5-Viruses in Chicken and Duck Embryos

Infected cell types in the chicken and duck embryos were determined by IHC staining for AIV. No differences were observed in antigen expressing cell types between the two poultry species or HPAI H5-viruses, but several differences could be observed between the investigated organs. In all tissues, endothelial cells were most abundantly stained followed by mononuclear cells (macrophages and lymphocytes) and/or Kupffer cells (liver), being stained positive in all tissues except for the heart. To a lesser extent, tissue-specific cells such as glia cells, neurons, respiratory epithelium, olfactory epithelium, cardiomyocytes, hepatocytes and serosal cells stained positive for the virus antigen. Virus antigen staining was almost absent in digestive epithelium of the intestine and limited in the respiratory epithelium of the nasal conchae, but staining in the submucosa could be detected (Figure S3).

### 3.6. Comparative Analysis of the Results

Comparative analysis was performed for the tested viruses relative to the most recent HPAI H5N1 virus, which caused the largest outbreak ever recorded in Europe [25]. The replication rate is analyzed from 6 h to 15 h post infection, 50% survival probability is displayed as time to death in hours post infection, and average virus antigen scores for the eight studied organs are summarized for the 24 h post infection time point in Table 3 (unprocessed data in Supplementary Table S8). No difference in IVPI score can be observed in chickens, but in ducks, the IVPI scores show a higher virulence for the H5N8-2016 and H5N6-2017 viruses compared to the H5N1-2021 reference strain. The H5N8-2014 virus showed relatively fast systemic virus dissemination in both ECEs and EDEs (IHC). Additionally, the H5N8-2014 virus replicated faster than the reference virus and had a shorter time to death in EDEs. The H5N8-2016 virus replicated slower in ECEs and time to death was longer, while no differences could be observed in replication rates and time to death for the EDEs compared to the reference virus. Furthermore, the H5N8-2016 virus showed relatively fast systemic virus dissemination in both ECEs and EDEs. The H5N6-2017 virus had a relatively slower replication rate, but no difference could be observed in the time to death compared to the reference virus in both ECEs and EDEs. Systemic virus dissemination of the H5N6-2017 virus was faster in ECEs, but no difference in virus dissemination could be observed in EDEs compared to the reference virus. Lastly, the H5N8-2020 virus replicated slower, had a slower time to death, and had a slower systemic virus dissemination in both ECEs and EDEs. Overall, the in ovo models indicated relatively fast replication combined with an average time to death and slower systemic virus dissemination for the H5N1-2021 virus compared to the other HPAI viruses.

## 4. Discussion

In this study, we developed novel ex vivo models to determine the virulence of HPAI H5-viruses for risk assessment for the poultry sector. We investigated five recent HPAI H5-viruses isolated in the Netherlands during the subsequent epizootics and compared the results from the ex vivo models to the IVPI scores obtained for these viruses. The IVPI score of the HPAI H5N1-2021 virus for chickens and ducks was determined in this study, whereas the IVPI scores for the other viruses were determined previously [19,20] (Table 2). ECE and EDE models were used to measure virus replication in the allantoic fluid, time to death and average virus antigen score in several organs. Currently, sampling of the allantoic fluid is performed by incubating the eggs at 4 °C for several hours (which kills and sediments the embryo), opening the egg shell and removing the chorioallantoic membrane. This method is not suitable for follow up samples, does not allow for the immediate extraction of the sample and may result in large egg-to-egg differences. We attached cannulas to the allantoic sac in order to directly sample at the desired time point and collect multiple samples from the same individual greatly reducing egg-to-egg differences. Furthermore, sampling time points can be optimized to distinguish minute differences between HPAI viruses. The age of the embryos was optimized to minimize egg-to-egg differences for the virus replication and time to death measurements while maintaining sufficient development of the embryo to harvest individual organs for IHC. Fourteen-day-old chicken and duck embryos were used in this study. The maturation of the embryos’ innate and adaptive immune system occurs throughout the development of the embryo. Fourteen-day-old chicken embryos have developed T-cells, B-cells and macrophages but the embryos are immunocompetent only just prior to hatching [44]. The development of duck embryos is slower (hatching after 28 days) than the development of chicken embryos (hatching after 21 days); therefore, the immune response of the 14-day-old ECEs and EDEs is likely limited. Egg survival monitored by candling is challenging in further-developed embryos since mortality can only be confirmed after sedimentation of the embryo which occurs several hours postmortem. Therefore, we used a heart rate monitor to accurately determine the time to death of the embryos increasing the objectivity and reducing false negative and false-positive mortality times.

In chickens, the five investigated HPAI H5-viruses all reached the maximum IVPI score of 3.00. However, the genetic distance between the 2.3.4.4c (H5N8-2014) and 2.3.4.4b (H5N8-2016, H5N6-2017, H5N8-2020 and H5N1-2021) subclades is relatively large (Figure S1), and the IVPI may not have sufficient resolution to detect the differences between highly virulent viruses. Interestingly, significant differences between the HPAI H5-viruses were detected using the chicken in ovo model in replication rate, time to death and average virus antigen score. This may suggest that the resolution of the chicken in ovo model exceeds that of the IVPI (Table S8). In the chicken in ovo model, the replication rate was higher and time to death was shorter for the HPAI H5N1-2021 virus than for the earlier viruses H5N8-2020 and H5N8-2016. The H5N1-2021 virus also has a higher replication rate than the H5N6-2017 virus, although no significant difference was measured for the time to death. Interestingly, the replication rate and time to death of the H5N1-2021 virus are similar to that of the H5N8-2014 virus, which belongs to another subclade. In the duck in ovo model, the H5N1-2021 virus has a higher replication rate then the H5N6-2017 and H5N8-2020 HPAI viruses. Similar replication rates are measured for the H5N8-2016 virus, and a higher replication rate is measured for the H5N8-2014 virus. A similar pattern is observed for the time to death in the duck in ovo model with the exception of the H5N6-2017 HPAI virus, which showed no significant difference with the H5N1-2021 virus. These results may suggest that the HPAI H5-virus evolved towards lower virulence for chickens and ducks between 2014 and 2020, but then increased in virulence for the H5N1-2021 virus. The largest difference in chicken replication rate compared to duck replication rate was observed for the H5N8-2016 and H5N6-2017 viruses which also had the highest IVPI scores in Pekin ducks. This shows replication rate is an important factor of virulence, but since replication rate in the duck in ovo model is also high for other HPAI viruses, virulence is likely influenced by multiple factors.

The spread of the virus in the embryo tissues was studied using IHC analysis. Tissue tropism in ECEs and EDEs was systemic and highly abundant in the well-perfused organs. In situ virus detection by IHC is not standard for IVPI experiments and was only studied for the H5N6-2017 virus [37]. In adult Pekin ducks, the H5N6-2017 virus showed a slight reduction in antigen score in all tissues compared to chickens, most notably in the intestine. The in ovo model showed similar results for the H5N6-2017 virus indicating a reduction in virus antigen score in all tissues for ducks compared to chickens. Natural infections and attachment patterns of H5N8-2016 and H5N8-2020 for several Anseriformes species showed increased enterotropism compared to the H5N8-2014 virus, and a high level of neurotropism was observed for the H5N8-2020 virus [45,46]. The increase in enterotropism was not observed in the in ovo models. However, virus antigen levels in the EDE cerebrum were lower for the H5N8-2014, H5N8-2016 and H5N6-2017 viruses than virus antigen levels in other tissues, while for the H5N8-2020 and H5N1-2021 viruses, similar levels of virus antigen could be observed in the EDE cerebrum compared to the other organs. This may indicate an increase in neurotropism in Pekin ducks for the H5N8-2020 (similarly to previous findings [45,46]) and H5N1-2021 viruses compared to the other HPAI viruses in this study. Surprisingly, only the H5N8-2020 virus showed a significantly lower systemic virus antigen dissemination than the H5N1-2021 virus, which is unexpected from the relatively fast replication rate (Table 3). Overall, these results indicate that in ovo models can identify changes in tissue tropism, which can offer valuable insights in virus replication and dissemination.

The virulence of a virus can be affected by many factors including replication, cytopathogenic effect, receptor binding, immune evasion and stability of the virus. Some of these important aspects of virulence can be measured in the in ovo models, while other factors are likely not included. The in ovo models will not be suitable to directly replace the IVPI experiment, but can provide additional information on the virulence of novel viruses for poultry, and to obtain more insight in the factors determining the virulence. In this study, only a limited number of H5-viruses were tested in the in ovo models, while multiple subtypes and genotypes circulated during the subsequent epizootics [5,21,22,23,24]. To study the evolution of virulence for poultry over the years, more viruses will have to be assessed including different subtypes and genotypes. Although the main genetic differences between the viruses used in this study were observed on the PB2, PB1 and NA segments, the mechanism underlying the differences in virulence for the H5-viruses is not well understood. Further studies will be required to provide more insight in the genetic determinants of virulence of H5-viruses. The viruses tested in this study were isolated from chickens or ducks, and the origin of the viruses may have affected the measured virulence.

Although the combination of multiple measurements in the in ovo models arguably provides the most accurate prediction of virulence, measuring the replication rate in the allantoic fluid is costly and requires optimization of the sampling time points. The in ovo time to death model is most suitable for large scale assessment of poultry virulence, since it requires minimal optimization of sampling time points, is easy to perform and is less costly than the other in ovo model measurements. The average virus antigen expression provides valuable information on systemic dissemination of the virus, but the benefit of cell-specific virus antigen scoring was limited for HPAI viruses. Therefore, we suspect similar results on virus dissemination can be obtained by studying fewer tissues. In a previous study, the in ovo virulence of LPAI and HPAI viruses differed greatly in the time to death and number of affected organs [29]. Since our model can distinguish the small differences in HPAI virulence in both chickens and ducks, we expect differences in virulence between LPAI and HPAI viruses can also be detected using the in ovo model presented in this study. The ex vivo models developed in this study have lower costs, do not make use of adult animals, and are less time consuming than the IVPI. This will facilitate the testing of larger sets of viruses, in particular, novel viruses detected in wild birds, to assess the risk for the poultry sector. Monitoring changes in HPAI virulence for poultry facilitates the annotation of genetic virulence factors, surveillance of host drift and rapid identification of HPAI infected animals with or without overt clinical disease. The results of this study show that ex vivo models are highly valuable tools for risk assessment of HPAI viruses, allowing for a more targeted approach for HPAI surveillance benefiting both public health and animal health.

## Figures and Tables

**Table 1:** Genetic background and general information of recent HPAI epizootic H5-viruses in this study.

Virus Isolate	EPI Number	Clade 2.3.4.4	Poultry Species Virus Was Isolated from	Differences between Swab Isolate and Second Egg Passage
A/chicken/Netherlands/14015531/2014	EPI_ISL_18600237	Group c	Chicken	PB2-R574K, PA-F451S, NA-A332V
A/duck/Netherlands/16014829-001005/2016	EPI_ISL_529179	Group b	Duck	PB2-V344A
A/Duck/Netherlands/17017236-001-005/2017	EPI_ISL_18600770	Group b	Duck	PB2-I30L
A/chicken/Netherlands/20016597-026030/2020	EPI_ISL_8650948	Group b	Chicken	-
A/chicken/Netherlands/21037287-006010/2021	EPI_ISL_9856775	Group b—genogroup AB	Chicken	-

**Table 2:** IVPI scores of recent HPAI epizootic H5-viruses in this study.

Virus Isolate	Abbreviation	IVPI Chicken	IVPI Pekin Duck
A/chicken/Netherlands/14015531/2014	H5N8-2014 [19]	3.00	1.87
A/duck/Netherlands/16014829-001005/2016	H5N8-2016 [19]	3.00	2.99
A/Duck/Netherlands/17017236-001-005/2017	H5N6-2017 [19]	2.99	3.00
A/chicken/Netherlands/20016597-026030/2020	H5N8-2020 [20]	2.98	1.74
A/chicken/Netherlands/21037287-006010/2021	H5N1-2021	3.00	1.96

**Table 3:** Fold change in IVPI- and in ovo-determined virulence from the H5N1-2021 reference virus (^a^) for chickens and Pekin ducks. Replication rate is summarized from 6 h to 15 h post infection. The 50% survival probability is displayed as time to death in hours post infection. Average IHC scores for the eight studied organs are calculated at 24 h post infection. Significantly different groups are depicted by * significantly different from reference, ** significantly different from reference and from * *p* < 0.05. (**) not significantly different from H5N6-2017. (*) significantly different at 28 h post infection.

	IVPI Chicken	Replication Rate Chicken	Time to Death Chicken	IHC Chicken	IVPI Pekin Duck	Replication Rate Pekin Duck	Time to Death Pekin Duck	IHC Pekin Duck
H5N8-2014	1.00	1.06	0.96	2.14 (**)	0.95	1.09 *	0.92 *	1.75 **
H5N8-2016	1.00	0.78 *	1.05 *	1.49 *	1.53	0.94	0.96	1.30 *
H5N6-2017	1.00	0.72 *	1.00	1.49 *	1.53	0.82 *	0.97	1.00
H5N8-2020	0.99	0.70 *	1.05 *	0.57 (*)	0.89	0.60 **	1.11 *	0.50 *
H5N1-2021 ^a^	1.00	1.00	1.00	1.00	1.00	1.00	1.00	1.00

## Data Availability

The data presented in this study are made available in this article and the provided Supplementary Materials.

## Supplementary Materials

The following supporting information can be downloaded at: https://www.mdpi.com/article/10.3390/v16040563/s1, Figure S1: The 26-gauge cannula attachment to embryonated chicken egg. Opening is closed with nail polish. Figure S2: Phylogenetic trees with the strains used in this study and a random selection of AI strains from the different European outbreak seasons (2014 to 2022) inferred using maximum likelihood (ML) methods. Trees are provided for each of the 8 segments. Tips shaded by outbreak season. Additional strains were obtained from the GISAID database (see acknowledgement, Table S9). Figure S3: Mean virus antigen score (immunohistochemistry) in embryonic chicken and Pekin duck organs inoculated with five HPAI H5 viruses. Organs are collected at 20 h, 24 h and 28 h post infection *n* = 5. Table S1: Replication in chicken embryo allantoic fluid significance determined by linear regression broken stick model for 0–6 h post infection and 6–15 h post infection. Table S2: Replication in duck embryo allantoic fluid significance determined by linear regression broken stick model for 0–6 h post infection and 6–15 h post infection. Table S3: The Cox model was used to measure the effect of the differences between chicken embryo survival times of the five HPAI H5-viruses using a Tukey-corrected pairwise comparison. Table S4: The Cox model was used to measure the effect of the differences between duck embryo survival times of the five HPAI H5-viruses using a Tukey-corrected pairwise comparison. Table S5: Organ-specific method for immunohistochemistry (IHC) and cell type scoring. Table S6: Chicken embryo virus antigen scores in different organs were grouped according to the best fitting model of the factor analysis followed by a Tukey-corrected pairwise comparison. Table S7: Chicken embryo virus antigen scores in different organs were grouped according to the best fitting model of the factor analysis followed by a Tukey-corrected pairwise comparison. Table S8: Summary of the largest differences measured for the in ovo models and IVPI in absolute values. Replication rate is summarized from 6 h to 15 h post infection. The 50% survival probability is displayed as time to death in hours post infection. Average IHC scores for the eight studied organs are calculated at 24 h post infection. Table S9: GISAID accession numbers.

## References

[1] Adlhoch C., Gossner C., Koch G., Brown I., Bouwstra R., Verdonck F., Penttinen P., Harder T. (2014). Comparing introduction to Europe of highly pathogenic avian influenza viruses A (H5N8) in 2014 and A (H5N1) in 2005. Eurosurveillance.

[2] Napp S., Majó N., Sánchez-Gónzalez R., Vergara-Alert J. (2018). Emergence and spread of highly pathogenic avian influenza A (H5N8) in Europe in 2016–2017. Transbound. Emerg. Dis..

[3] Poen M.J., Venkatesh D., Bestebroer T.M., Vuong O., Scheuer R.D., Oude Munnink B.B., de Meulder D., Richard M., Kuiken T., Koopmans M.P. (2019). Co-circulation of genetically distinct highly pathogenic avian influenza A clade 2.3. 4.4 (H5N6) viruses in wild waterfowl and poultry in Europe and East Asia, 2017–2018. Virus Evol..

[4] King J., Schulze C., Engelhardt A., Hlinak A., Lennermann S.-L., Rigbers K., Skuballa J., Staubach C., Mettenleiter T.C., Harder T. (2020). Novel HPAIV H5N8 reassortant (clade 2.3. 4.4 b) detected in Germany. Viruses.

[5] Adlhoch C., Fusaro A., Gonzales J.L., Kuiken T., Marangon S., Niqueux É., Staubach C., EFSA, ECDC, EURL (2022). Scientific report: Avian influenza overview December 2021–March 2022. EFSA J..

[6] Rijks J.M., Leopold M.F., Kühn S., in ‘t Veld R., Schenk F., Brenninkmeijer A., Lilipaly S.J., Ballmann M.Z., Kelder L., de Jong J.W. (2022). Mass Mortality Caused by Highly Pathogenic Influenza A (H5N1) Virus in Sandwich Terns, the Netherlands, 2022. Emerg. Infect. Dis..

[7] Bordes L., Vreman S., Heutink R., Roose M., Venema S., Pritz-Verschuren S.B., Rijks J.M., Gonzales J.L., Germeraad E.A., Engelsma M. (2023). Highly pathogenic avian influenza H5N1 virus infections in wild red foxes (Vulpes vulpes) show neurotropism and adaptive virus mutations. Microbiol. Spectr..

[8] Vreman S., Kik M., Germeraad E., Heutink R., Harders F., Spierenburg M., Engelsma M., Rijks J., van den Brand J., Beerens N. (2023). Zoonotic Mutation of Highly Pathogenic Avian Influenza H5N1 Virus Identified in the Brain of Multiple Wild Carnivore Species. Pathogens.

[9] World Health Organization, Organisation mondiale de la Santé (2022). Antigenic and genetic characteristics of zoonotic influenza A viruses and development of candidate vaccine viruses for pandemic preparedness—Caractéristiques génétiques et antigéniques des virus grippaux A zoonotiques et mise au point de virus vaccinaux candidats pour se préparer à une pandémie. Wkly. Epidemiol. Rec. Relev. Épidémiologique Hebd..

[10] Beerens N., Germeraad E.A., Venema S., Verheij E., Pritz-Verschuren S.B., Gonzales J.L. (2021). Comparative pathogenicity and environmental transmission of recent highly pathogenic avian influenza H5 viruses. Emerg. Microbes Infect..

[11] Pantin-Jackwood M.J., Costa-Hurtado M., Shepherd E., DeJesus E., Smith D., Spackman E., Kapczynski D.R., Suarez D.L., Stallknecht D.E., Swayne D.E. (2016). Pathogenicity and transmission of H5 and H7 highly pathogenic avian influenza viruses in mallards. J. Virol..

[12] Pantin-Jackwood M.J., Costa-Hurtado M., Bertran K., DeJesus E., Smith D., Swayne D.E. (2017). Infectivity, transmission and pathogenicity of H5 highly pathogenic avian influenza clade 2.3. 4.4 (H5N8 and H5N2) United States index viruses in Pekin ducks and Chinese geese. Vet. Res..

[13] WOAH, Manual of Diagnostic Tests and Vaccines for Terrestrial Animals. https://www.woah.org/en/what-we-do/standards/codes-and-manuals/terrestrial-manual-online-access/.

[14] Webster R.G., Rott R. (1987). Influenza virus A pathogenicity: The pivotal role of hemagglutinin. Cell.

[15] Böttcher-Friebertshäuser E., Garten W., Matrosovich M., Klenk H.D. (2014). The hemagglutinin: A determinant of pathogenicity. Influenza Pathog. Control.

[16] Abdelwhab E.-S.M., Veits J., Mettenleiter T.C. (2013). Genetic changes that accompanied shifts of low pathogenic avian influenza viruses toward higher pathogenicity in poultry. Virulence.

[17] in ‘t Veld R., Richard M., Fouchier R., Monne I., Kuiken T., EMC, IZSVe (2017). Mechanisms and risk factors for mutation from low to highly pathogenic avian influenza virus. EFSA Support. Publ..

[18] Lee D.-H., Criado M.F., Swayne D.E. (2021). Pathobiological origins and evolutionary history of highly pathogenic avian influenza viruses. Cold Spring Harb. Perspect. Med..

[19] Beerens N., Heutink R., Pritz-Verschuren S., Germeraad E., Bergervoet S., Harders F., Bossers A., Koch G. (2019). Genetic relationship between poultry and wild bird viruses during the highly pathogenic avian influenza H5N6 epidemic in the Netherlands, 2017–2018. Transbound. Emerg. Dis..

[20] Engelsma M., Heutink R., Harders F., Germeraad E.A., Beerens N. (2022). Multiple Introductions of Reassorted Highly Pathogenic Avian Influenza H5Nx Viruses Clade 2.3. 4.4 b Causing Outbreaks in Wild Birds and Poultry in The Netherlands, 2020–2021. Microbiol. Spectr..

[21] Brown I., Mulatti P., Smietanka K., Staubach C., Willeberg P., Adlhoch C., Candiani D., EFSA, ECDC, EURL (2017). Scientific report on the avian influenza overview October 2016–August 2017. EFSA J..

[22] Adlhoch C., Brouwer A., Kuiken T., Mulatti P., Smietanka K., Staubach C., Muñoz Guajardo I., EFSA, ECDC, EURL (2018). Scientific report: Avian influenza overview February–May 2018. EFSA J..

[23] EFSA (2014). Highly pathogenic avian influenza A subtype H5N8. EFSA J..

[24] Adlhoch C., Fusaro A., Gonzales J.L., Kuiken T., Marangon S., Niqueux É., Staubach C., EFSA, ECDC, EURL (2022). Scientific report: Avian influenza overview May–September 2021. EFSA J..

[25] Adlhoch C., Fusaro A., Gonzales J.L., Kuiken T., Marangon S., Niqueux É., Staubach C., EFSA, ECDC, EURL (2022). Scientific report: Avian influenza overview June–September 2022. EFSA J..

[26] Vigeveno R.M., Poen M.J., Parker E., Holwerda M., de Haan K., van Montfort T., Lewis N.S., Russell C.A., Fouchier R.A., de Jong M.D. (2020). Outbreak severity of highly pathogenic avian influenza A (H5N8) viruses is inversely correlated to polymerase complex activity and interferon induction. J. Virol..

[27] Youk S.-S., Leyson C.M., Seibert B.A., Jadhao S., Perez D.R., Suarez D.L., Pantin-Jackwood M.J. (2021). Mutations in PB1, NP, HA, and NA contribute to increased virus fitness of H5N2 highly pathogenic avian influenza virus clade 2.3. 4.4 in chickens. J. Virol..

[28] Bortz E., Westera L., Maamary J., Steel J., Albrecht R.A., Manicassamy B., Chase G., Martínez-Sobrido L., Schwemmle M., García-Sastre A. (2011). Host-and strain-specific regulation of influenza virus polymerase activity by interacting cellular proteins. MBio.

[29] Seekings A.H., Howard W.A., Nuñéz A., Slomka M.J., Banyard A.C., Hicks D., Ellis R.J., Nuñéz-García J., Hartgroves L.C., Barclay W.S. (2020). The emergence of H7N7 highly pathogenic avian influenza virus from low pathogenicity avian influenza virus using an in ovo embryo culture model. Viruses.

[30] Parvin R., Schinkoethe J., Grund C., Ulrich R., Bönte F., Behr K.P., Voss M., Samad M.A., Hassan K.E., Luttermann C. (2020). Comparison of pathogenicity of subtype H9 avian influenza wild-type viruses from a wide geographic origin expressing mono-, di-, or tri-basic hemagglutinin cleavage sites. Vet. Res..

[31] de Bruin A., Spronken M.I., Bestebroer T.M., Fouchier R.A., Richard M. (2022). Reduced replication of highly pathogenic avian influenza virus in duck endothelial cells compared to chicken endothelial cells is associated with stronger antiviral responses. Viruses.

[32] Bouwstra R., Koch G., Heutink R., Harders F., Van Der Spek A., Elbers A., Bossers A. (2015). Phylogenetic analysis of highly pathogenic avian influenza A (H5N8) virus outbreak strains provides evidence for four separate introductions and one between-poultry farm transmission in the Netherlands, November 2014. Eurosurveillance.

[33] Reed L.J., Muench H. (1938). A simple method of estimating fifty per cent endpoints. Am. J. Epidemiol..

[34] Katoh K., Toh H. (2010). Parallelization of the MAFFT multiple sequence alignment program. Bioinformatics.

[35] Nguyen L.-T., Schmidt H.A., Von Haeseler A., Minh B.Q. (2015). IQ-TREE: A fast and effective stochastic algorithm for estimating maximum-likelihood phylogenies. Mol. Biol. Evol..

[36] Yu G., Smith D.K., Zhu H., Guan Y., Lam T.T.Y. (2017). ggtree: An R package for visualization and annotation of phylogenetic trees with their covariates and other associated data. Methods Ecol. Evol..

[37] Vreman S., Bergervoet S.A., Zwart R., Stockhofe-Zurwieden N., Beerens N. (2022). Tissue tropism and pathology of highly pathogenic avian influenza H5N6 virus in chickens and Pekin ducks. Res. Vet. Sci..

[38] Therneau T. (2015). Mixed effects Cox models. CRAN Repos..

[39] R Core Team R: A Language and Environment for Statistical Computing. R Foundation for Statistical Computing. http://www.R-project.org/.

[40] Bates D., Mächler M., Bolker B., Walker S. (2015). Fitting linear mixed-effects models using lme4. J. Stat. Softw..

[41] Therneau T. (2023). A Package for Survival Analysis in R (R Package Version 3.5-0). CRAN Repos..

[42] Harrell F.E., Harrell M.F.E. (2019). Package ‘hmisc’. CRAN Repos..

[43] Lenth R., Singmann H., Love J., Buerkner P., Herve M. (2020). Emmeans: Estimated marginal means, aka least-squares means, R package version 1. 5. 1. CRAN Repos..

[44] Hincke M.T., Da Silva M., Guyot N., Gautron J., McKee M.D., Guabiraba-Brito R., Réhault-Godbert S. (2019). Dynamics of structural barriers and innate immune components during incubation of the avian egg: Critical interplay between autonomous embryonic development and maternal anticipation. J. Innate Immun..

[45] Caliendo V., Leijten L., Begeman L., Poen M.J., Fouchier R.A., Beerens N., Kuiken T. (2020). Enterotropism of highly pathogenic avian influenza virus H5N8 from the 2016/2017 epidemic in some wild bird species. Vet. Res..

[46] Caliendo V., Leijten L., van de Bildt M., Germeraad E., Fouchier R.A., Beerens N., Kuiken T. (2022). Tropism of highly pathogenic avian influenza H5 viruses from the 2020/2021 epizootic in wild ducks and geese. Viruses.

