# Supplementary material for: In Ovo Models to Predict Virulence of Highly Pathogenic Avian Influenza H5-Viruses for Chickens and Ducks
Source: Viruses. 2024 Apr 4;16(4):563. doi: 10.3390/v16040563 (PMC11053719; doi:10.3390/v16040563)
Supplement: Supplementary file 1 [file viruses-16-00563-s001.zip › Supplementary.pdf]

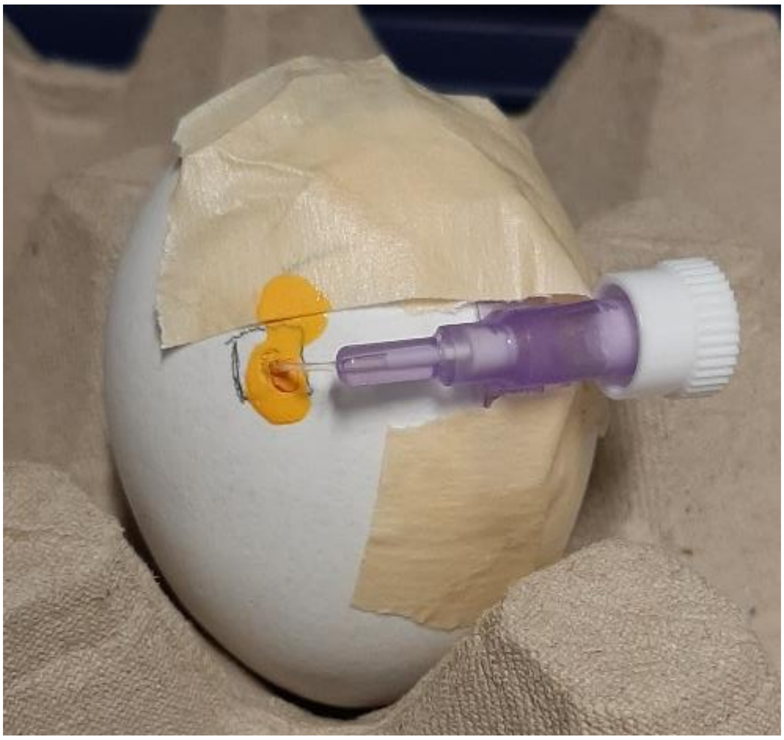

Figure S1: 26 Gauge cannula attachment to embryonated chicken egg. Opening is closed with nail polish.

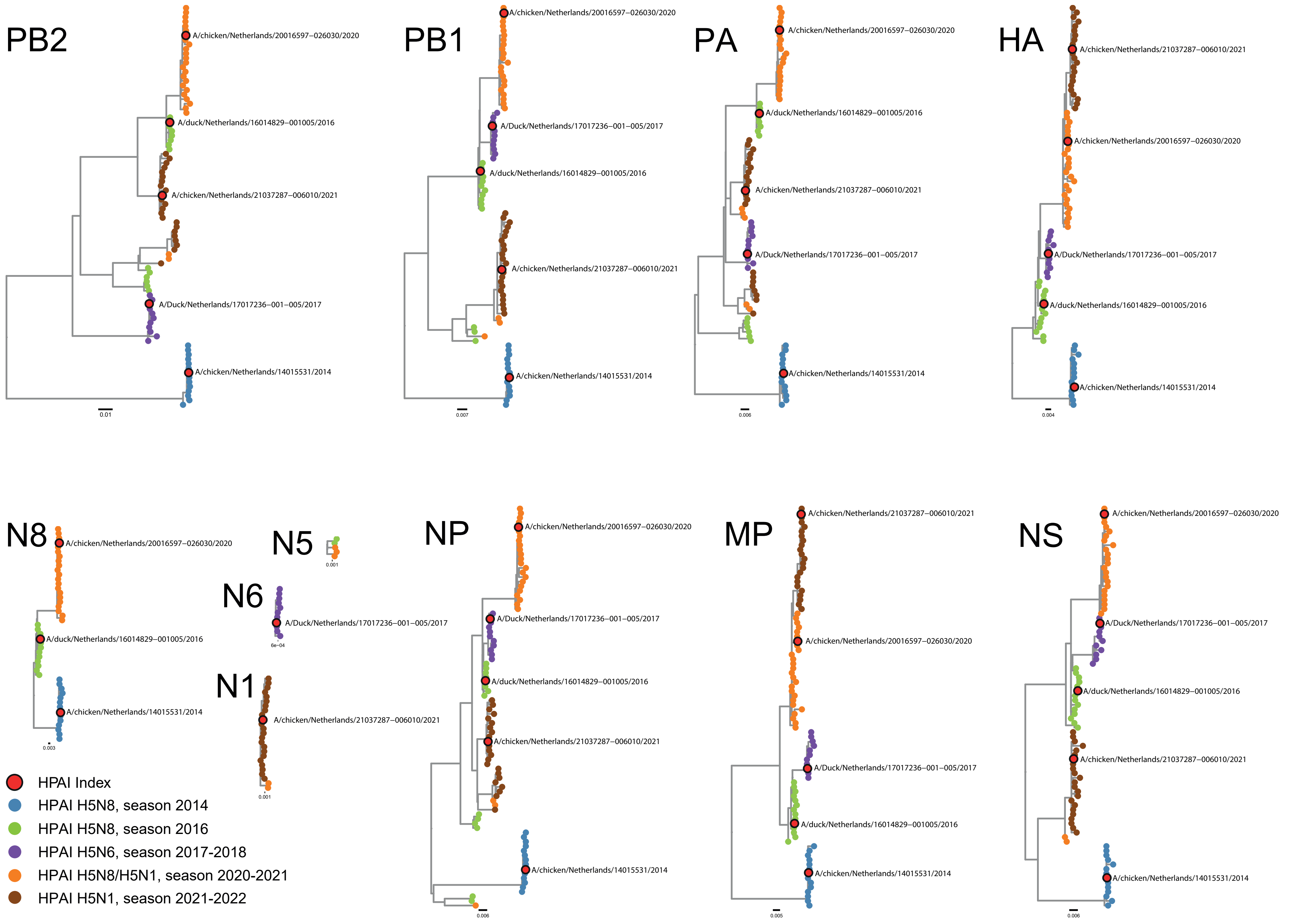

Figure S2: Phylogenetic trees with the strains used in this study and a random selection of AI strains from the different European outbreak seasons (2014 to 2022) inferred using maximum likelihood (ML) methods. Trees are provided for each of the 8 segments. Tips shaded by outbreak season. Additional strains were obtained from the GISAID database (see acknowledgement table S9).

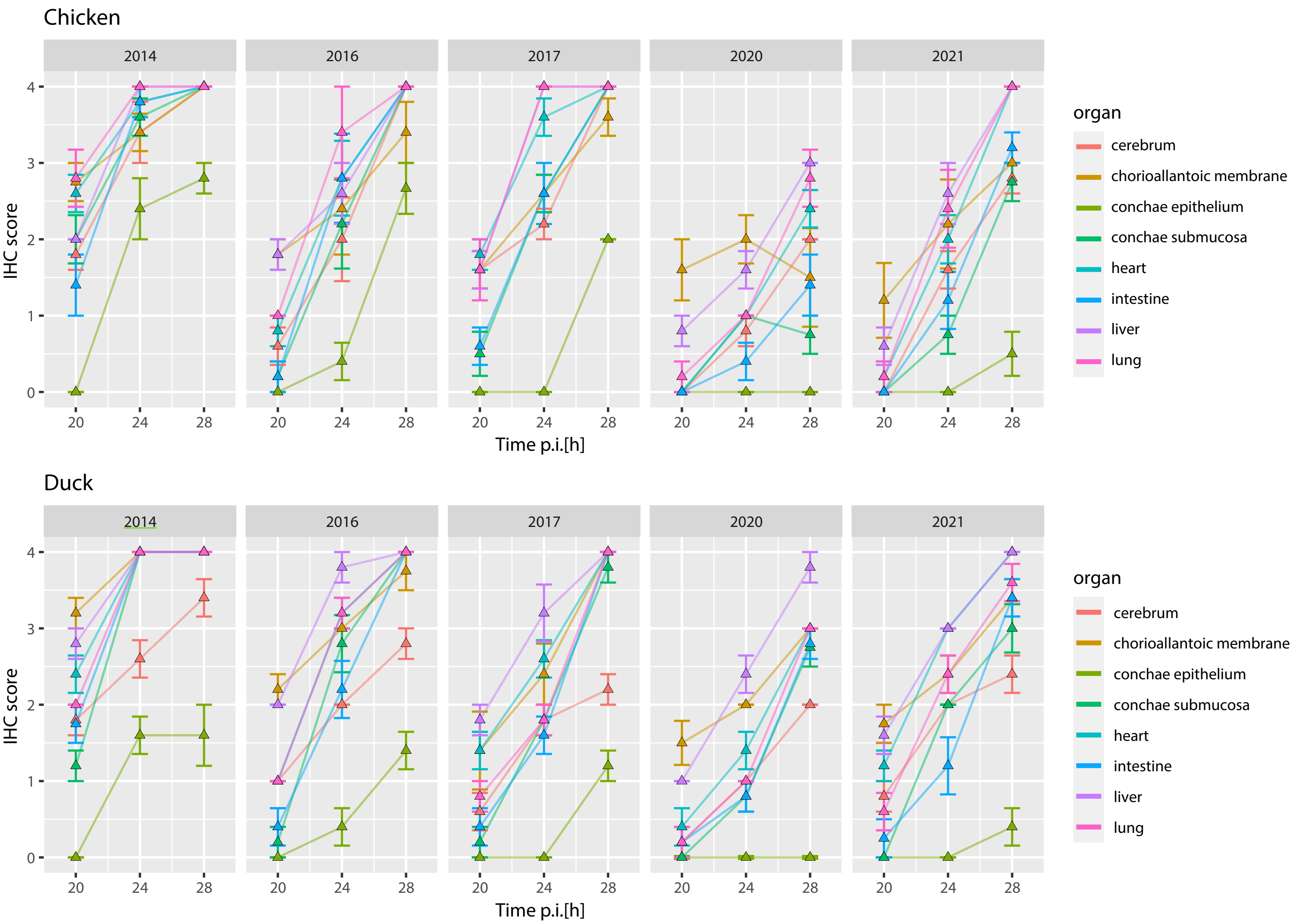

Figure S3: Mean virus antigen score (immunohistochemistry) in embryonic chicken and Pekin duck organs inoculated with five HPAI H5 viruses. Organs are collected at 20 hours , 24 hours and 28 hours post infection *n*=5.

Table S1: Replication in chicken embryo allantoic fluid significance determined by linear regression broken stick model for 0-6 hours post infection and 6-15 hours post infection.

Model	Virus comparison	t-ratio	p-value
**Chicken 0h-6h replication**	virus2014 - virus2016	3.636	0.0030
**Chicken 0h-6h replication**	virus2014 - virus2017	2.740	0.0635
**Chicken 0h-6h replication**	virus2014 - virus2020	3.560	0.0040
**Chicken 0h-6h replication**	virus2014 - virus2021	2.615	0.0918
**Chicken 0h-6h replication**	virus2016 - virus2017	-0.865	1.0000
**Chicken 0h-6h replication**	virus2016 - virus2020	0.003	1.0000
**Chicken 0h-6h replication**	virus2016 - virus2021	-0.986	1.0000
**Chicken 0h-6h replication**	virus2017 - virus2020	0.851	1.0000
**Chicken 0h-6h replication**	virus2017 - virus2021	-0.121	1.0000
**Chicken 0h-6h replication**	virus2020 - virus2021	-0.969	1.0000
**Chicken 6h-15h replication**	virus2014 - virus2016	5.555	<.0001
**Chicken 6h-15h replication**	virus2014 - virus2017	6.783	<.0001
**Chicken 6h-15h replication**	virus2014 - virus2020	7.165	<.0001
**Chicken 6h-15h replication**	virus2014 - virus2021	1.274	1.0000
**Chicken 6h-15h replication**	virus2016 - virus2017	1.186	1.0000
**Chicken 6h-15h replication**	virus2016 - virus2020	1.673	0.9492
**Chicken 6h-15h replication**	virus2016 - virus2021	-4.136	0.0004
**Chicken 6h-15h replication**	virus2017 - virus2020	0.511	1.0000
**Chicken 6h-15h replication**	virus2017 - virus2021	-5.322	<.0001
**Chicken 6h-15h replication**	virus2020 - virus2021	-5.726	<.0001

Table S2: Replication in duck embryo allantoic fluid significance determined by linear regression broken stick model for 0-6 hours post infection and 6-15 hours post infection.

Model	Virus comparison	t-ratio	p-value
**Duck 0h-6h replication**	virus2014 - virus2016	0.706	1.0000
**Duck 0h-6h replication**	virus2014 - virus2017	0.477	1.0000
**Duck 0h-6h replication**	virus2014 - virus2020	1.059	1.0000
**Duck 0h-6h replication**	virus2014 - virus2021	2.023	0.4358
**Duck 0h-6h replication**	virus2016 - virus2017	-0.226	1.0000
**Duck 0h-6h replication**	virus2016 - virus2020	0.360	1.0000
**Duck 0h-6h replication**	virus2016 - virus2021	1.295	1.0000
**Duck 0h-6h replication**	virus2017 - virus2020	0.582	1.0000
**Duck 0h-6h replication**	virus2017 - virus2021	1.520	1.0000
**Duck 0h-6h replication**	virus2020 - virus2021	0.910	1.0000
**Duck 6h-15h replication**	virus2014 - virus2016	3.444	0.0062
**Duck 6h-15h replication**	virus2014 - virus2017	6.304	<.0001
**Duck 6h-15h replication**	virus2014 - virus2020	11.221	<.0001
**Duck 6h-15h replication**	virus2014 - virus2021	2.020	0.4390
**Duck 6h-15h replication**	virus2016 - virus2017	2.812	0.0510
**Duck 6h-15h replication**	virus2016 - virus2020	7.716	<.0001
**Duck 6h-15h replication**	virus2016 - virus2021	-1.400	1.0000
**Duck 6h-15h replication**	virus2017 - virus2020	4.957	<.0001
**Duck 6h-15h replication**	virus2017 - virus2021	-4.213	0.0003
**Duck 6h-15h replication**	virus2020 - virus2021	-9.090	<.0001

Table S3: The Cox model was used to measure the effect of the differences between chicken embryo survival times of the five HPAI H5-viruses using a Tukey-corrected pairwise comparison .

Virus comparison	t-ratio	p-value
**virus2014 - virus2016**	5.197	<.0001
**virus2014 - virus2017**	2.639	0.0634
**virus2014 - virus2020**	5.144	<.0001
**virus2014 - virus2021**	2.625	0.0659
**virus2016 - virus2017**	-2.907	0.0300
**virus2016 - virus2020**	0.034	1.0000
**virus2016 - virus2021**	-2.954	0.0261
**virus2017 - virus2020**	2.875	0.0329
**virus2017 - virus2021**	0.005	1.0000
**virus2020 - virus2021**	-2.930	0.0280

Table S4: The Cox model was used to measure the effect of the differences between duck embryo survival times of the five HPAI H5-viruses using a Tukey-corrected pairwise comparison.

Virus comparison	t-ratio	p-value
**virus2014 - virus2016**	4.732	<.0001
**virus2014 - virus2017**	4.970	<.0001
**virus2014 - virus2020**	10.319	<.0001
**virus2014 - virus2021**	6.494	<.0001
**virus2016 - virus2017**	0.295	0.9984
**virus2016 - virus2020**	7.725	<.0001
**virus2016 - virus2021**	2.658	0.0604
**virus2017 - virus2020**	7.657	<.0001
**virus2017 - virus2021**	2.430	0.1073
**virus2020 - virus2021**	-6.218	<.0001

Table S5: Organ specific method for immunohistochemistry (IHC) and cell type scoring.

**Organ**	**Scoring method**
Cerebrum IHC score 1	< 5 positive cells on average of 5 microscopic fields with objective 10x
Cerebrum IHC score 2	> 5 -30 positive cell on average of 5 microscopic fields, multifocal staining not coalescent
Cerebrum IHC score 3	Multifocal to coalescent staining of cells, including neuropil (< 50% of neuropil)
Cerebrum IHC score 4	Multifocal to coalescent staining of cells, including neuropil (> 50% of neuropil)
Conchae submucosa IHC score 1	Multifocal staining of individual blood vessel
Conchae submucosa IHC score 2	Multifocal to coalescing staining of individual blood vessels, mainly superficial in mucosa
Conchae submucosa IHC score 3	Diffuse staining of individual blood vessels in superficial and deep mucosa and staining of white blood cells within vessels
Conchae submucosa IHC score 4	Diffuse staining of submucosa and large vessels
Conchae epithelial cells IHC score 1	Staining of few epithelial cells (< 5 foci), mostly individual cells with objective 20x
Conchae epithelial cells IHC score 2	Multifocal staining of individual epithelial cells, but also clusters of 2-5 cells together (< 10 foci)
Conchae epithelial cells IHC score 3	Multifocal staining of individual epithelial cells, but also clusters of 2-5 cells together (> 10 foci)
Conchae epithelial cells IHC score 4	Not evaluated, no tissues with this expression
Lung IHC score 1	< 5 positive cells/foci on average of 5 microscopic fields with objective 10x
Lung IHC score 2	> 5 -30 positive cells/foci on average of 5 microscopic field, multifocal staining not coalescent
Lung IHC score 3	Diffuse staining of endothelial cells
Lung IHC score 4	Diffuse staining of endothelial cells, individual staining cells are difficult to recognize
Heart IHC score 1	< 10 positive cells/foci on average of 5 microscopic fields with objective 10x
Heart IHC score 2	> 10 -50 positive cells/foci on average of 5 microscopic field, multifocal staining not coalescent
Heart IHC score 3	Diffuse staining of endothelial cells and also cardiomyocytes
Heart IHC score 4	Diffuse staining of endothelial cells and also cardiomyocytes, individual staining cells are difficult to recognize
Liver IHC score 1	< 10 positive cells/foci on average of 5 microscopic fields with objective 20x
Liver IHC score 2	> 10 -50 positive cells/foci on average of 5 microscopic field, multifocal staining not coalescent
Liver IHC score 3	Diffuse staining of cells lining sinusoids and in sinusoids
Liver IHC score 4	Diffuse staining of cells lining sinusoids and in sinusoids, also staining of hepatocytes individual staining cells are difficult to recognize

Intestine IHC score 1	< 10 positive cells/foci on average of 5 microscopic fields with objective 20x
Intestine IHC score 2	> 10 -50 positive cells/foci on average of 5 microscopic field, multifocal staining not coalescent, also serosal cells
Intestine IHC score 3	Diffuse staining of serosal cells
Intestine IHC score 4	Diffuse staining of serosal cells, extending to muscular layer
Chorion allantoic membrane IHC score 1	< 5 positive cells/foci on average of 5 microscopic fields with objective 10x
Chorion allantoic membrane IHC score 2	> 5 -30 positive cells/foci on average of 5 microscopic field, multifocal staining not coalescent
Chorion allantoic membrane IHC score 3	Diffuse staining of endothelial cells
Chorion allantoic membrane IHC score 4	Diffuse staining of endothelial cells, individual staining cells are difficult to recognize

Table S6: Chicken embryo virus antigen scores in different organs were grouped according to the best fitting model of the factor analysis followed by a Tukey corrected pairwise comparison.

Model	Virus comparison	t-ratio	p-value
**Chicken 20h**	virus2014 - virus2016	4.069	0.0017
**Chicken 20h**	virus2014 - virus2017	2.456	0.1194
**Chicken 20h**	virus2014 - virus2020	5.678	<.0001
**Chicken 20h**	virus2014 - virus2021	5.019	0.0001
**Chicken 20h**	virus2016 - virus2017	-1.481	0.5802
**Chicken 20h**	virus2016 - virus2020	1.917	0.3236
**Chicken 20h**	virus2016 - virus2021	1.932	0.3156
**Chicken 20h**	virus2017 - virus2020	3.223	0.0190
**Chicken 20h**	virus2017 - virus2021	3.014	0.0327
**Chicken 20h**	virus2020 - virus2021	0.382	0.9953
**Chicken 24h**	virus2014 - virus2016	3.790	0.0039
**Chicken 24h**	virus2014 - virus2017	2.174	0.2080
**Chicken 24h**	virus2014 - virus2020	6.862	<.0001
**Chicken 24h**	virus2014 - virus2021	6.595	<.0001
**Chicken 24h**	virus2016 - virus2017	-1.615	0.4958
**Chicken 24h**	virus2016 - virus2020	3.998	0.0021
**Chicken 24h**	virus2016 - virus2021	3.022	0.0320
**Chicken 24h**	virus2017 - virus2020	5.219	<.0001
**Chicken 24h**	virus2017 - virus2021	4.545	0.0004
**Chicken 24h**	virus2020 - virus2021	-1.521	0.5547
**Chicken 28h**	virus2014 - virus2016	0.012	1.0000
**Chicken 28h**	virus2014 - virus2017	0.150	0.9999
**Chicken 28h**	virus2014 - virus2020	6.105	<.0001
**Chicken 28h**	virus2014 - virus2021	1.821	0.3745
**Chicken 28h**	virus2016 - virus2017	0.118	1.0000
**Chicken 28h**	virus2016 - virus2020	5.450	<.0001
**Chicken 28h**	virus2016 - virus2021	1.588	0.5127
**Chicken 28h**	virus2017 - virus2020	5.976	<.0001
**Chicken 28h**	virus2017 - virus2021	1.680	0.4563
**Chicken 28h**	virus2020 - virus2021	-4.238	0.0010

Table S7: Chicken embryo virus antigen scores in different organs were grouped according to the best fitting model of the factor analysis followed by a Tukey corrected pairwise comparison.

Model	Virus comparison	t-ratio	p-value
**Duck 20h**	virus2014 - virus2016	4.766	0.0001
**Duck 20h**	virus2014 - virus2017	5.583	<.0001
**Duck 20h**	virus2014 - virus2020	7.898	<.0001
**Duck 20h**	virus2014 - virus2021	6.128	<.0001
**Duck 20h**	virus2016 - virus2017	0.817	0.9244
**Duck 20h**	virus2016 - virus2020	3.132	0.0217
**Duck 20h**	virus2016 - virus2021	1.362	0.6541
**Duck 20h**	virus2017 - virus2020	2.315	0.1545
**Duck 20h**	virus2017 - virus2021	0.545	0.9822
**Duck 20h**	virus2020 - virus2021	-1.770	0.4003
**Duck 24h**	virus2014 - virus2016	5.174	<.0001
**Duck 24h**	virus2014 - virus2017	8.851	<.0001
**Duck 24h**	virus2014 - virus2020	12.800	<.0001
**Duck 24h**	virus2014 - virus2021	8.306	<.0001
**Duck 24h**	virus2016 - virus2017	3.677	0.0045
**Duck 24h**	virus2016 - virus2020	7.625	<.0001
**Duck 24h**	virus2016 - virus2021	3.132	0.0217
**Duck 24h**	virus2017 - virus2020	3.949	0.0019
**Duck 24h**	virus2017 - virus2021	-0.545	0.9822
**Duck 24h**	virus2020 - virus2021	-4.494	0.0003
**Duck 28h**	virus2014 - virus2016	2.043	0.2588
**Duck 28h**	virus2014 - virus2017	1.770	0.4003
**Duck 28h**	virus2014 - virus2020	6.672	<.0001
**Duck 28h**	virus2014 - virus2021	3.813	0.0029
**Duck 28h**	virus2016 - virus2017	-0.272	0.9988
**Duck 28h**	virus2016 - virus2020	4.630	0.0002
**Duck 28h**	virus2016 - virus2021	1.770	0.4003
**Duck 28h**	virus2017 - virus2020	4.902	0.0001
**Duck 28h**	virus2017 - virus2021	2.043	0.2588
**Duck 28h**	virus2020 - virus2021	-2.860	0.0444

Table S8: Summary of the largest differences measured for the i*n ovo* models and IVPI in absolute values. Replication rate is summarized from 6 hours to 15 hours post infection. The 50% survival probability is displayed as time to death in hours post infection. Average IHC scores for the eight studied organs is calculated at 24 hours post infection.

Virus	IVPI chicken	Replication rate chicken	Time to death chicken	IHC chicken	IVPI Pekin duck	Replication rate Pekin duck	Time to death Pekin duck	IHC Pekin duck
H5N8-2014	3	0,354	28,44	3,75	1,87	0,42	24,53	3,5
H5N8-2016	3	0,261	31,21	2,6	2,99	0,364	25,77	2,6
H5N6-2017	2,99	0,241	29,62	2,6	3	0,317	25,86	2
H5N8-2020	2,98	0,232	31,23	1	1,74	0,232	29,84	1
H5N1-2021	3	0,333	29,62	1,75	1,96	0,387	26,78	2

Table S9: GISAID accession numbers.

We gratefully acknowledge the authors, originating and submitting laboratories of the sequences from GISAID's EpiFlu™ Database on which this research is based. The list is detailed below.
All submitters of data may be contacted directly via www.gisaid.org

Isolate-ID	Isolate name	Country	Collection date	Originating Lab	Submitting Lab	Authors
EPI_ISL_167140	A/turkey/Germany-MV/R2472/2014	Germany	2014-11-04		Friedrich-Loeffler-Institut	
EPI_ISL_167904	A/duck/England/36254/14	United Kingdom	2014-11-14	Animal and Plant Health Agency (APHA)	Animal and Plant Health Agency (APHA)	Hanna, Amanda; Ellis, Richard; Ceeraz, Vanessa; Seekings, James; Londt, Brandon; Brookes, Sharon; Banks, Jill; Essen, Stephen; Brown, Ian
EPI_ISL_168075	A/chicken/Netherlands/14015531/2014	Netherlands	2014-11-15	Wageningen Bioveterinary Research	Wageningen Bioveterinary Research	Heutink, Rene; Harders, Frank; Verschuren-Pritz, Sylvia; Bossers, Alex; Koch, Guus; Bouwstra, Ruth
EPI_ISL_331223	A/Duck/England/1279/2014	United Kingdom	2014-11-16	Animal and Plant Health Agency (APHA)	Animal and Plant Health Agency (APHA)	Puranik,Anita; Warren,Caroline; Mahmood,Sahar; Thomas,Saumya; Byrne,Alexander; Ramsay,Andrew; Everett,Helen; Skinner,Paul; Núñez,Alejandro; Watson,Samantha; Slomka,Marek; Brown,Ian; Brookes,Sharon
EPI_ISL_174349	A/chicken/Netherlands/14015766/2014	Netherlands	2014-11-19	Wageningen Bioveterinary Research	Wageningen Bioveterinary Research	Heutink, Rene; Harders, Frank; Verschuren-Pritz, Sylvia; Bossers, Alex; Koch, Guus; Bouwstra, Ruth
EPI_ISL_174350	A/Chicken/Netherlands/14015824/2014	Netherlands	2014-11-20	Wageningen Bioveterinary Research	Wageningen Bioveterinary Research	Heutink, Rene; Harders, Frank; Verschuren-Pritz, Sylvia; Bossers, Alex; Koch, Guus; Bouwstra, Ruth
EPI_ISL_174351	A/duck/Netherlands/14015989/2014	Netherlands	2014-11-21	Wageningen Bioveterinary Research	Wageningen Bioveterinary Research	Heutink, Rene; Harders, Frank; Verschuren-Pritz, Sylvia; Bossers, Alex; Koch, Guus; Bouwstra, Ruth
EPI_ISL_181095	A/chicken/Netherlands/emc-3/2014	Netherlands	2014-11-21			Verhagen,J.H.; Van der Jeugd,H.P.; Nolet,B.A.; Vuong,O.; Majoor,F.; De Vries,P.P.; Kharitonov,S.; Kuiken,T.; Fouchier,R.A.M.
EPI_ISL_181093	A/eurasian wigeon/Netherlands/1/2014	Netherlands	2014-11-24			Verhagen,J.H.; Van der Jeugd,H.P.; Nolet,B.A.; Vuong,O.; Majoor,F.; De Vries,P.P.; Kharitonov,S.; Kuiken,T.; Fouchier,R.A.M.
EPI_ISL_181094	A/eurasian wigeon/Netherlands/2/2014	Netherlands	2014-11-24			Verhagen,J.H.; Van der Jeugd,H.P.; Nolet,B.A.; Vuong,O.; Majoor,F.; De Vries,P.P.; Kharitonov,S.; Kuiken,T.; Fouchier,R.A.M.
EPI_ISL_174352	A/chicken/Netherlands/14016437/2014	Netherlands	2014-11-29	Wageningen Bioveterinary Research	Wageningen Bioveterinary Research	Heutink, Rene; Harders, Frank; Verschuren-Pritz, Sylvia; Bossers, Alex; Koch, Guus; Bouwstra, Ruth
EPI_ISL_169851	A/stork/Germany-MV/R24/2015	Germany	2014-12-01		Friedrich-Loeffler-Institut	
EPI_ISL_169350	A/turkey/Italy/14VIR7898-10/2014	Italy	2014-12-15	Istituto Zooprofilattico Sperimentale Delle Venezie	Istituto Zooprofilattico Sperimentale Delle Venezie	Luca,Tassoni; Silvia,Ormelli; Alessia,Schivo; Alice,Fusaro; Isabella,Monne; Giovanni,Cattoli
EPI_ISL_169351	A/turkey/Germany-NI/R3372/2014	Germany	2014-12-15		Friedrich-Loeffler-Institut	
EPI_ISL_169351	A/turkey/Germany-NI/R3372/2014	Germany	2014-12-15		Friedrich-Loeffler-Institut	
EPI_ISL_239434	A/tufted_duck/Switzerland/V246-L02001/2016	Switzerland	2016-11-01	Institut für Virologie und Immunologie - Bundesamt für Lebensmittelsicherheit und Veterinärwesen	Friedrich-Loeffler-Institut	
EPI_ISL_268675	A/T_Dk/NL-Zeewolde/16013976-001-003/2016	Netherlands	2016-11-09	Wageningen Bioveterinary Research	Wageningen Bioveterinary Research	Beerens, Nancy; Heutink, Rene; Harders, Frank; Verschuren-Pritz, Sylvia; Bossers, Alex; Koch, Guus; Bergervoet, Saskia
EPI_ISL_436135	A/chicken/Germany-MV/AR9528-L02970/2016	Germany	2016-11-18	Landesamt für Landwirtschaft, Lebensmittelsicherheit und Fischerei (LALLF)	Friedrich-Loeffler-Institut	
EPI_ISL_238895	A/Chicken/Sweden/SVA161122KU0453/SZ0209318/2016	Sweden	2016-11-21	Swedish Veterinary Agency (SVA)	Swedish Veterinary Agency (SVA)	
EPI_ISL_529179	A/duck/Netherlands/16014829-001005/2016	Netherlands	2016-11-25	Wageningen Bioveterinary Research	Wageningen Bioveterinary Research	Beerens, Nancy; Harders, Frank; Verschuren-Pritz, Sylvia; Bossers, Alex; Heutink, Rene
EPI_ISL_271712	A/Goose/Hungary/63743/2016	Hungary	2016-12-11	National Food Chain Safety Office Veterinary Diagnostic Directorate Laboratory for Molecular Biology	Danam.Vet.Molbiol	Adam, Dan
EPI_ISL_268650	A/Eur_Wig/NL-West Graftdijk/16015746-003/2016	Netherlands	2016-12-12	Wageningen Bioveterinary Research	Wageningen Bioveterinary Research	Beerens, Nancy; Heutink, Rene; Harders, Frank; Verschuren-Pritz, Sylvia; Bossers, Alex; Koch, Guus; Bergervoet, Saskia
EPI_ISL_240893	A/swan/Germany-SN/R10645/2016	Germany	2016-12-13		Friedrich-Loeffler-Institut	
EPI_ISL_268635	A/Eur_Wig/NL-Akkrum/16015817-003/2016	Netherlands	2016-12-13	Wageningen Bioveterinary Research	Wageningen Bioveterinary Research	Beerens, Nancy; Heutink, Rene; Harders, Frank; Verschuren-Pritz, Sylvia; Bossers, Alex; Koch, Guus; Bergervoet, Saskia
EPI_ISL_268641	A/Eur_Wig/NL-Gouda/16015824-001/2016	Netherlands	2016-12-13	Wageningen Bioveterinary Research	Wageningen Bioveterinary Research	Beerens, Nancy; Heutink, Rene; Harders, Frank; Verschuren-Pritz, Sylvia; Bossers, Alex; Koch, Guus; Bergervoet, Saskia

]

EPI_ISL_4064461	A/duck/France/161298/2016	France	2016-12-16			Briand,F.-X.; Niqueux,E.; Schmitz,A.; Martenot,C.; Cherbonnel,M.; Kerbrat,F.; Chatel,M.; Quenault,H.; Beven,V.; Leroux,A.; Hirchaud,E.; Lucas,P.; Blanchard,Y.; Grasland,B.
EPI_ISL_4064466	A/duck/France/161449/2016	France	2016-12-22			Briand,F.-X.; Niqueux,E.; Schmitz,A.; Martenot,C.; Cherbonnel,M.; Kerbrat,F.; Chatel,M.; Quenault,H.; Beven,V.; Leroux,A.; Hirchaud,E.; Lucas,P.; Blanchard,Y.; Grasland,B.
EPI_ISL_243085	A/wigeon/Italy/16VIR9616-3/2016	Italy	2016-12-29	Istituto Zooprofilattico Sperimentale Delle Venezie	Istituto Zooprofilattico Sperimentale Delle Venezie	Silvia,Ormelli; Sabrina,Marciano; Alessia,Schivo; Annalisa,Salviato; Adelaide,Milani; Gianpiero,Zamperini; Bianca,Zecchin; Alice,Fusaro; Calogero,Terregino; Isabella,Monne
EPI_ISL_5034851	A/chicken/Poland/115/2016(H5N8)	Poland	2016-12-29	National Veterinary Research Institut Poland, PIWet-PIB	National Veterinary Research Institut Poland, PIWet-PIB	Swieton E., Smietanka K.
EPI_ISL_287906	A/Duck/Netherlands/17017236-001-005/2017	Netherlands	2017-12-07	Wageningen Bioveterinary Research	Wageningen Bioveterinary Research	Beerens, Nancy; Heutink, Rene; Harders, Frank; Verschuren-Pritz, Sylvia; Bossers, Alex; Koch, Guus; Bergervoet, Saskia
EPI_ISL_332433	A/Bird/Netherlands/17017775-035-039/2017	Netherlands	2017-12-15	Wageningen Bioveterinary Research	Wageningen Bioveterinary Research	Beerens, Nancy; Heutink, Rene; Harders, Frank; Verschuren-Pritz, Sylvia; Bossers, Alex; Koch, Guus; Bergervoet, Saskia
EPI_ISL_292225	A/canada_goose/England/AV58_18OPpoolEP1/2018	United Kingdom	2018-01-05	Animal and Plant Health Agency (APHA)	Animal and Plant Health Agency (APHA)	Seekings, James; Ellis, Richard; Brookes, Sharon M; Reid, Scott; Essen, Stephen; Lewis, Nicola; Brown, Ian H
EPI_ISL_332436	A/Peacock/Netherlands/18000887-006/2018	Netherlands	2018-01-20	Wageningen Bioveterinary Research	Wageningen Bioveterinary Research	Beerens, Nancy; Heutink, Rene; Harders, Frank; Verschuren-Pritz, Sylvia; Bossers, Alex; Koch, Guus; Bergervoet, Saskia
EPI_ISL_302823	A/Great black-backed gull/Netherlands/1/2018	Netherlands	2018-01-23	Erasmus Medical Center	Erasmus Medical Center	Poen,MJ; Bestebroer,TM; Vuong,O; Scheuer,RD; Kelder,L; Fouchier,RAM
EPI_ISL_18795093	A/Common buzzard/SVA180307SZ0416/KU000389/K-2018	Sweden	2018-03-07	Swedish Veterinary Agency (SVA)	Swedish Veterinary Agency (SVA)	"Siamak,Zohari"
EPI_ISL_305417	A/Domestic_Duck/Netherlands/EMC-6/2018	Netherlands	2018-03-13	Erasmus Medical Center	Erasmus Medical Center	Poen,MJ; Bestebroer,TM; De Meulder,D; Vuong,O; Scheuer,RD; Netherlands Food and Consumer Product Safety Authority,NVWA;Koopmans,MPG; Fouchier,RAM
EPI_ISL_305454	A/chicken/Germany-SH/AR164-L02543/2018	Germany	2018-03-19		Friedrich-Loeffler-Institut	
EPI_ISL_306989	A/turkey/Germany-SH/AR185-L02549/2018	Germany	2018-03-19		Friedrich-Loeffler-Institut	
EPI_ISL_313226	A/white stork/Germany-NI/AR251/2018	Germany	2018-04-01	Niedersaechsisches Landesgesundheitsamt	Friedrich-Loeffler-Institut	
EPI_ISL_332441	A/Mallard/Netherlands/18012508-017/2018	Netherlands	2018-08-24	Wageningen Bioveterinary Research	Wageningen Bioveterinary Research	Beerens, Nancy; Heutink, Rene; Harders, Frank; Verschuren-Pritz, Sylvia; Bossers, Alex; Koch, Guus; Bergervoet, Saskia
EPI_ISL_654827	A/goose/Omsk/30003/2020	Russian Federation	2020-09-03	State Research Center of Virology and Biotechnology (VECTOR)	State Research Center of Virology and Biotechnology (VECTOR)	Natalia,Goncharova; Ivan,Susloparov; Natalia,Kolosova; Alexey,Danilenko; Juliya,Bulanovich; Vasiliy,Marchenko; Alexander,Ryzhikov
EPI_ISL_654824	A/duck/Saratov/29804/2020	Russian Federation	2020-09-15	State Research Center of Virology and Biotechnology (VECTOR)	State Research Center of Virology and Biotechnology (VECTOR)	Natalia,Goncharova; Ivan,Susloparov; Natalia,Kolosova; Alexey,Danilenko; Juliya,Bulanovich; Vasiliy,Marchenko; Alexander,Ryzhikov
EPI_ISL_5863358	A/mallard/Georgia/DT-22360/2020	Georgia	2020-10-02	Erasmus Medical Center	Royal Veterinary College	Fouchier, R.A.M.; Vuong, O.; Scheuer, R.D.; Lopes, S.; Lewis, N.S.
EPI_ISL_603134	A/Eurasian Wigeon/Netherlands/4/2020	Netherlands	2020-10-16	Erasmus Medical Center	Erasmus Medical Center	
EPI_ISL_603132	A/chicken/Netherlands/20016597-026030/2020	Netherlands	2020-10-28	Wageningen Bioveterinary Research	Wageningen Bioveterinary Research	Beerens, Nancy; Harders, Frank; Verschuren-Pritz, Sylvia; Roose, Marit; Germeraad, Evelien; Engelsma, Marc; Bossers, Alex; Heutink, Rene
EPI_ISL_8650948	A/chicken/Netherlands/20016597-026030/2020	Netherlands	2020-10-28	Wageningen Bioveterinary Research	Wageningen Bioveterinary Research	Beerens, Nancy; Harders, Frank; Pritz-Verschuren, Sylvia; Roose, Marit; Venema, Sandra; Germeraad, Evelien; Engelsma, Marc; Heutink, Rene
EPI_ISL_1139025	A/barnacle goose/Netherlands/20016974-002/2020	Netherlands	2020-11-02	Wageningen Bioveterinary Research	Wageningen Bioveterinary Research	Beerens, Nancy; Harders, Frank; Pritz-Verschuren, Sylvia; Roose, Marit; Germeraad, Evelien; Engelsma, Marc; Bossers, Alex; Heutink, Rene
EPI_ISL_1139082	A/barnacle goose/Netherlands/20016896-011/2020	Netherlands	2020-11-02	Wageningen Bioveterinary Research	Wageningen Bioveterinary Research	Beerens, Nancy; Harders, Frank; Pritz-Verschuren, Sylvia; Roose, Marit; Germeraad, Evelien; Engelsma, Marc; Bossers, Alex; Heutink, Rene
EPI_ISL_1139041	A/greylag goose/Netherlands/20017064-002/2020	Netherlands	2020-11-03	Wageningen Bioveterinary Research	Wageningen Bioveterinary Research	Beerens, Nancy; Harders, Frank; Pritz-Verschuren, Sylvia; Roose, Marit; Germeraad, Evelien; Engelsma, Marc; Bossers, Alex; Heutink, Rene
EPI_ISL_710512	A/whistling_duck/England/035643/2020	United Kingdom	2020-11-19	Animal and Plant Health Agency (APHA)	Animal and Plant Health Agency (APHA)	

EPI_ISL_956409	A/common_teal/Italy/20VIR7439-190/2020	Italy	2020-11-28	Istituto Zooprofilattico Sperimentale delle Venezie, EU/OIE/Reference Laboratory and FAO Reference Centre for AI and ND	Istituto Zooprofilattico Sperimentale Delle Venezie	Zecchin, B.; Fusaro, A.; Milani, A.; Schivo, A.; Salviato, A.; Pastori, A.; Zamperin, G.; Monne, I.; Terregino, C.
EPI_ISL_779129	A/turkey/Poland/464/2020(H5N8)	Poland	2020-12-01	National Veterinary Research Institut Poland, PIWet-PIB	National Veterinary Research Institut Poland, PIWet-PIB	Swieton E., Smietanka K.
EPI_ISL_18718161	A/duck/France/PPNL-20P017941/2020	France	2020-12-10	Ploufragan-Plouzane-Niort Laboratory	Ploufragan-Plouzane-Niort Laboratory	Briand,F.-x., Niqueux,E., Schmitz,A., Martenot,C., Cherbonnel,M., Massin,P., Busson,R., Guillemoto,C., Pierre,I., Louboutin,K., Souchaud,F., Allee,C., Quenault,H., Beven,V., Leroux,A., Lucas,P., Hirchaud,E., Van de Wiele,A., Blanchard,Y., Scoizec,A., LeBouquin,S.; Grasland,B.
EPI_ISL_1039238	A/chicken/Astrakhan/321-09/2020	Russian Federation	2020-12-12	State Research Center of Virology and Biotechnology (VECTOR)	State Research Center of Virology and Biotechnology (VECTOR)	Natalia,Goncharova; Ivan,Susloparov; Natalia,Kolosova; Alexey,Danilenko; Juliya,Bulanovich; Vasiliy,Marchenko; Alexander,Ryzhikov
EPI_ISL_1122425	A/chicken/England/043315/2020	United Kingdom	2020-12-15	Animal and Plant Health Agency (APHA)	Animal and Plant Health Agency (APHA)	
EPI_ISL_846623	A/swan/Poland/MB141/2020(H5N8)	Poland	2020-12-16	National Veterinary Research Institut Poland, PIWet-PIB	National Veterinary Research Institut Poland, PIWet-PIB	Swieton, E.; Smietanka, K.
EPI_ISL_1123351	A/duck/England/043628/2020	United Kingdom	2020-12-18	Animal and Plant Health Agency (APHA)	Animal and Plant Health Agency (APHA)	
EPI_ISL_1665267	A/mute_swan/Slovenia/1914-20_21VIR959-5/2020	Slovenia	2020-12-24	Istituto Zooprofilattico Sperimentale delle Venezie, EU/OIE/Reference Laboratory and FAO Reference Centre for AI and ND	Istituto Zooprofilattico Sperimentale Delle Venezie	Slavec, B.; Racnik, J.; Zorman Rojs, Olga.; Zecchin, B.; Fusaro, A.; Pastori, A.; Schivo, A.; Salviato, A.; Monne, I.; Terregino, C.
EPI_ISL_7622862	A/Anser_brachyrhynchus/Belgium/151/2020	Belgium	2020-12-26	Sciensano - Animal Infectious Diseases	Sciensano, Department of Animal Infectious Diseases	Van Borm, Steven; Vandenbussche, Frank; Roupie, Virginie; Lambrecht, Benedicte; Steensels, Mieke
EPI_ISL_7778769	A/pheasant/Finland/499_21VIR7689-1/2021	Finland	2021-01-01	Finnish Food Authority	Istituto Zooprofilattico Sperimentale Delle Venezie	Tammiranta, N.; Kantala, T.; Laamanen, I.; Gadd, T.; Zecchin, B.; Fusaro, A.; Schivo, A.; Salviato, A.; Palumbo, E.; Milani, A.; Giussani, E.; Pastori, A.; Monne, I.; Terregino, C.
EPI_ISL_775267	A/turkey/Netherlands/21020942-001005/2021	Netherlands	2021-01-04	Wageningen Bioveterinary Research	Wageningen Bioveterinary Research	Beerens, Nancy; Harders, Frank; Pritz-Verschuren, Sylvia; Roose, Marit; Germeraad, Evelien; Engelsma, Marc; Bossers, Alex; Heutink, Rene
EPI_ISL_5095321	A/turkey/Germany-NI/AI00429/2021	Germany	2021-01-08	Lebensmittel- und Veterinärinstitut Oldenburg - Standort Veterinärinstitut	Friedrich-Loeffler-Institut	
EPI_ISL_943555	A/Chicken/Sweden/SVA210117SZ0004/KN011321/2021	Sweden	2021-01-16	Swedish Veterinary Agency (SVA)	Swedish Veterinary Agency (SVA)	
EPI_ISL_7778760	A/mute_swan/Estonia/TA2106615_21VIR7512-4/2021	Estonia	2021-03-04	Estonian Veterinary and Food Laboratory	Istituto Zooprofilattico Sperimentale Delle Venezie	Nurmoja, I.; Vilem, A.; Juurik, T.; Zecchin, B.; Fusaro, A.; Schivo, A.; Salviato, A.; Palumbo, E.; Milani, A.; Giussani, E.; Pastori, A.; Monne, I.; Terregino, C.
EPI_ISL_1697187	A/chicken/Czech Republic/6542-1/2021	Czech Republic	2021-04-03	State Veterinary Institute Prague	State Veterinary Institute Prague	Nagy,A;Cernikova,L;Stara,M
EPI_ISL_18458048	A/chicken/Lithuania/4525_21VIR3369-3/2021	Lithuania	2021-04-25	Lithuanian National Food and Veterinary Risk Assessment Institute (NFVRAI)	Istituto Zooprofilattico Sperimentale Delle Venezie	Pileviciene, S.;Janeliunas, Z.;Zecchin, B.;Pastori, A.;Fusaro, A.;Schivo, A.;Salviato, A.;Palumbo, E.;Giussani, E.;Monne, I.;Terregino, C.
EPI_ISL_7753173	A/Eurasian wigeon/Germany-SH/AI05956/2021	Germany	2021-10-14	Landeslabor Schleswig-Holstein	Friedrich-Loeffler-Institut	
EPI_ISL_9856775	A/chicken/Netherlands/21037287-006010/2021	Netherlands	2021-10-25	Wageningen Bioveterinary Research	Wageningen Bioveterinary Research	Beerens, Nancy; Harders, Frank; Pritz-Verschuren, Sylvia; Roose, Marit; Venema, Sandra; Germeraad, Evelien; Engelsma, Marc; Heutink, Rene; Luca, Bordes
EPI_ISL_6101869	A/goose/Netherlands/21037720-001/2021	Netherlands	2021-10-28	Wageningen Bioveterinary Research	Wageningen Bioveterinary Research	Beerens, Nancy; Harders, Frank; Pritz-Verschuren, Sylvia; Roose, Marit; Germeraad, Evelien; Engelsma, Marc; Heutink, Rene
EPI_ISL_7054770	A/European herring gull/Sweden/SVA211116SZ0432/FB004518/M-2021	Sweden	2021-11-08	Swedish Veterinary Agency (SVA)	Swedish Veterinary Agency (SVA)	
EPI_ISL_7683079	A/mute swan/Netherlands/21039627-002/2021	Netherlands	2021-11-26	Wageningen Bioveterinary Research	Wageningen Bioveterinary Research	Beerens, Nancy; Harders, Frank; Pritz-Verschuren, Sylvia; Roose, Marit; Venema, Sandra; Germeraad, Evelien; Engelsma, Marc; Heutink, Rene
EPI_ISL_14760648	A/chicken/Italy/21VIR10382/2021	Italy	2021-11-29	Istituto Zooprofilattico Sperimentale delle Venezie, EU/OIE/Reference Laboratory and FAO Reference Centre for AI and ND	Istituto Zooprofilattico Sperimentale delle Venezie	Barbierato, G.; Zecchin, B.; Fusaro, A.; Schivo, A.; Salviato, A.; Palumbo, E.; Giussani, E.; Pastori, A.; Monne, I.; Terregino, C.
EPI_ISL_14760678	A/chicken/Italy/21VIR10637-2/2021	Italy	2021-12-04	Istituto Zooprofilattico Sperimentale delle Venezie, EU/OIE/Reference Laboratory and FAO Reference Centre for AI and ND	Istituto Zooprofilattico Sperimentale delle Venezie	Barbierato, G.; Zecchin, B.; Fusaro, A.; Schivo, A.; Salviato, A.; Palumbo, E.; Giussani, E.; Pastori, A.; Monne, I.; Terregino, C.

EPI_ISL_13370520	A/mute_swan/England/244574/2021	United Kingdom	2021-12-06	Animal and Plant Health Agency (APHA)	Animal and Plant Health Agency (APHA)	
EPI_ISL_18361460	A/grey heron/Croatia/132-b/2021	Croatia	2021-12-08	Croatian Veterinary Institute, Poultry Centre	Istituto Zooprofilattico Sperimentale delle Venezie	Savić, Vladimir; Pastori, Ambra; Zecchin, Bianca; Fusaro, Alice; Schivo, Alessia; Salviato, Annalisa; Palumbo, Elisa; Giussani, Edoardo; Monne, Isabella; Terregino, Calogero
EPI_ISL_14760774	A/chicken/Italy/21VIR11385-1/2021	Italy	2021-12-16	Istituto Zooprofilattico Sperimentale delle Venezie, EU/OIE/Reference Laboratory and FAO Reference Centre for AI and ND	Istituto Zooprofilattico Sperimentale delle Venezie	Barbierato, G.; Zecchin, B.; Fusaro, A.; Schivo, A.; Salviato, A.; Palumbo, E.; Giussani, E.; Pastori, A.; Monne, I.; Terregino, C.
EPI_ISL_14760849	A/turkey/Italy/21VIR11509/2021	Italy	2021-12-20	Istituto Zooprofilattico Sperimentale delle Venezie, EU/OIE/Reference Laboratory and FAO Reference Centre for AI and ND	Istituto Zooprofilattico Sperimentale delle Venezie	Barbierato, G.; Zecchin, B.; Fusaro, A.; Schivo, A.; Salviato, A.; Palumbo, E.; Giussani, E.; Pastori, A.; Monne, I.; Terregino, C.
EPI_ISL_13370629	A/Common_Buzzard/England/245285/2021	United Kingdom	2021-12-31	Animal and Plant Health Agency (APHA)	Animal and Plant Health Agency (APHA)	
EPI_ISL_11007535	A/swan/Slovenia/13_22VIR777-8/2022	Slovenia	2022-01-03	University of Ljubljana	Istituto Zooprofilattico Sperimentale delle Venezie	Slavec, B.; Ra?nik, J.; Krape?, U.; ?labravec, Z.; A?ko, J.; Cociancich, V.; Paller, T.; Vidrih, ?.; Rojs, O.Z.; Arseniev, S.; Groza, O.; Barbierato, G.; Zecchin, B.; Fusaro, A.; Schivo, A.; Salviato, A.; Palumbo, E.; Giussani, E.; Monne, I.; Terregino, C.
EPI_ISL_8694715	A/chicken/Croatia/7/2022	Croatia	2022-01-10	Croatian Veterinary Institute, Poultry Centre	Croatian Veterinary Institute	Savić, Vladimir; Pastori, Ambra; Zecchin, Bianca; Fusaro, Alice; Schivo, Alessia; Salviato, Annalisa; Palumbo, Elisa; Giussani, Edoardo; Monne, Isabella; Terregino, Calogero
EPI_ISL_11112340	A/Barnacle Goose/Netherlands/10/2022	Netherlands	2022-02-07	Erasmus Medical Center	Erasmus Medical Center	
EPI_ISL_11259294	A/chicken/Spain/452-1_22VIR2142-21/2022	Spain	2022-02-07	Laboratorio Central de Veterinaria	Istituto Zooprofilattico Sperimentale delle Venezie	Ruano, M.J.; Rocha, A.; Sanchez, A.; Aguero, M; Barbierato, G.; Zecchin, B.; Fusaro, A.; Schivo, A.; Salviato, A.; Palumbo, E.; Giussani, E.; Monne, I.; Terregino, C.
EPI_ISL_11560324	A/Graylag goose/Netherlands/2/20222	Netherlands	2022-03-06	Erasmus Medical Center	Erasmus Medical Center	
EPI_ISL_13990733	A/chicken/Spain/942-8_22VIR6312-34/2022	Spain	2022-03-08	Laboratorio Central de Veterinaria	Istituto Zooprofilattico Sperimentale delle Venezie	Ruano, M.J.; Rocha, A.; Sanchez, A.; Aguero, M; Barbierato, G.; Zecchin, B.; Fusaro, A.; Schivo, A.; Salviato, A.; Palumbo, E.; Giussani, E.; Monne, I.; Terregino, C.
EPI_ISL_13370916	A/chicken/England/033318/2022	United Kingdom	2022-03-27	Animal and Plant Health Agency (APHA)	Animal and Plant Health Agency (APHA)	
EPI_ISL_12215419	A/goose/Netherlands/22007407-002/2022	Netherlands	2022-04-15	Wageningen Bioveterinary Research	Wageningen Bioveterinary Research	Beerens, Nancy; Harders, Frank; Pritz-Verschuren, Sylvia; Roose, Marit; Venema, Sandra; Germeraad, Evelien; Engelsma, Marc; Heutink, Rene
EPI_ISL_14987487	A/common guillemot/Sweden/SVA220727SZ0354/FB002466/I-2022	Sweden	2022-07-27	Swedish Veterinary Agency (SVA)	Swedish Veterinary Agency (SVA)	
EPI_ISL_14497870	A/domestic_duck/England/105414/2022	United Kingdom	2022-08-07	Animal and Plant Health Agency (APHA)	Animal and Plant Health Agency (APHA)	
EPI_ISL_15038819	A/partridge/England/115171/2022	United Kingdom	2022-09-05	Animal and Plant Health Agency (APHA)	Animal and Plant Health Agency (APHA)	
